# Nitroxidative Stress, Cell—Signaling Pathways, and Manganese Porphyrins: Therapeutic Potential in Neuropathic Pain

**DOI:** 10.3390/ijms26052050

**Published:** 2025-02-26

**Authors:** Álvaro José Chávez Silva, Mário Sérgio Lima de Lavor

**Affiliations:** Department of Agricultural and Environmental Sciences, State University of Santa Cruz (UESC), Ilhéus 45662-900, BA, Brazil; alchasi91@gmail.com

**Keywords:** chronic pain, manganese porphyrin, neuropathic pain, oxidative stress, UPR

## Abstract

Neuropathic pain, a debilitating condition arising from somatosensory system damage, significantly impacts quality of life, leading to anxiety, self-mutilation, and depression. Oxidative and nitrosative stress, an imbalance between reactive oxygen and nitrogen species (ROS/RNS) and antioxidant defenses, plays a crucial role in its pathophysiology. While reactive species are essential for physiological functions, excessive levels can cause cellular component damage, leading to neuronal dysfunction and pain. This review highlights the complex interactions between reactive species, antioxidant systems, cell signaling, and neuropathic pain. We discuss the physiological roles of ROS/RNS and the detrimental effects of oxidative and nitrosative stress. Furthermore, we explore the potential of manganese porphyrins, compounds with antioxidant properties, as promising therapeutic agents to mitigate oxidative stress and alleviate neuropathic pain by targeting key cellular pathways involved in pain. Further research is needed to fully understand their therapeutic potential in managing neuropathic pain in human and non-human animals.

## 1. Introduction

Chronic pain is a major source of human suffering and disability [1]. Although pain itself and many diseases associated with chronic pain are not immediately fatal, they significantly reduce the quality of life of adults and animals worldwide. This impact is especially severe in patients with neuropathy, in whom sleep disorders, anxiety, and depression are frequent and intense, constituting a risk factor in suicide attempts [2,3]. Neuropathic pain is a specific type of chronic pain, described by persistent or intermittent spontaneous pain, and increased response to mechanical and thermal stimuli, either innocuous or noxious (allodynia or hyperalgesia, respectively), that arises from lesions to the somatosensory nervous system of peripheral or central origin [4], ectopic activity in sensorial fibers, and an imbalance of inhibitory and excitatory neurotransmitters relation, with the involvement of neuroinflammation and nitroxidative stress playing a pivotal role in its instauration and maintenance [5,6]. It is associated with the need for more prescription medications to obtain desired effects, which can lead to adverse effects due to increased doses and frequent visits to health professionals [7,8].

Although neuropathic pain is a multifaceted condition driven by several cellular and molecular mechanisms, those considered key to its development and perpetuation are neuroinflammation, glial cell activation, the release of proinflammatory cytokines, and oxidative stress [5], contributing to altered signaling pathways, which facilitates the expression of further inflammatory mediators and enhanced sensitivity to pain, ultimately, maintaining the cycle of pain where neuroinflammation and oxidative stress influence each other [6,9].

Oxidative stress is known as the imbalance between the cellular oxidative and antioxidant systems, and is widely recognized in chronic pain conditions, since excessive ROS production, exceeding the capacity of cellular antioxidant systems, can result in a dysfunction of the main cellular mitochondrial and endoplasmic reticulum (ER) systems, increasing ROS/PRN concentration during stress conditions [10,11,12,13].

Free radicals are molecules highly reactive with unpaired electrons in the outer valence, which makes them inherently chemically unstable and prone to react with surrounding molecules to achieve stabilization [14]. Free radicals can be produced exogenously from ionizing radiation, ozone, cigarette smoking, or by-products of environmental metabolisms. Endogenous sources, such as those derived from oxygen (reactive oxygen species, ROS) or nitrogen (reactive nitrogen species, RNS), are produced through enzymatical or non-enzymatical processes, for instance, in the electron transfer chain (ETC), phagocytosis, cytochrome P-450 system, and prostaglandin (PG) synthesis during normal neuronal functioning or during neuroinflammation processes associated with neuropathic pain, either accepting an electron from or donating an electron to other molecules, consequently, acting also as oxidants [15,16]. Such chemical behavior, when excessive, generates an environment suitable for damage on molecules such as DNA, carbohydrates, lipids, and proteins, leading to homeostasis disruption and cellular damage, and ultimately leading to apoptosis by lowering enzymatic and non-enzymatic antioxidants and compromising cell viability [17,18].

A great amount of information has been developed around free radical chemistry, leading to a better understanding of their biological significance, beyond the concept of harmful uncontrolled molecules. Importantly, it is known that free radicals originate in normal physiological conditions, and have several physiological functions in cellular growth, differentiation, migration, apoptosis, and necrosis, as well as defense against pathogens and species evolution [16,19] Additionally, increasing information sustains the role of reactive species in normal synaptic plasticity and cognitive functions such as memory and learning [20,21], and transducing agonistic modulation during neuropathic pain [22].

Several metalloporphyrins have been identified as potent catalysts for numerous redox reactions; particularly manganese porphyrins (MnPs), which have been found to be efficient antioxidant catalysts [23,24]. These substances dismute superoxide ions at some of the highest rates known for synthetic catalysts and have demonstrated therapeutic effects in conditions where increased ROS/PRN production is a key pathological factor [25], overcoming major conventional monotherapy approaches, usually unsuccessful and accompanied with cumulative adverse effects.

Research into the therapeutic potential of MnPs with antioxidant properties studied in disease models involving oxidative stress [26,27] and in models of chronic pain [28] considering their structural, chemical, pharmacokinetic, and pharmacodynamic properties, is necessary to elucidate the clinical uses of MnPs, based on understanding the pathways and mechanisms that underlie chronic pain of neuropathic origin.

This review examines the nature of ROS/RNS, endogenous antioxidant systems, nitroxidative signaling in neuropathic pain, and the potential of synthetic MnPs in the restitution of neuronal redox homeostasis, which is crucial for understanding scavengers’ therapeutic strategies, considering that neuropathic pain conditions still face significant limitation towards effective treatments on human and non-human animals.

## 2. Reactive Oxygen and Nitrogen Species

Molecular oxygen and nitrogen are undeniably significant in biology, being essential for proper metabolism, cellular function, and the physiological state of eustress in living organisms. While these molecules play critical roles in structural and functional processes, such as signal transduction, gene transcription and expression, and other cellular activities, they can also exert harmful effects on biomolecules in the form of reactive oxygen and nitrogen species, thereby illustrating their dual physiological and pathological nature [29].

Since most of reactive species’ effects rely on their compartmental concentration and their chemical nature, it is relevant to understand their main subcellular sources, allowing the compartmentalization of the reactive species gradients, redox-mediated regulation, and role in cell-signaling from several membrane receptors [19,30,31]. In this section, we discuss the chemistry and sources of ROS and RNS in the context of neuropathic pain.

### 2.1. Reactive Oxygen Species: Chemistry and Sources

**Figure 1:**
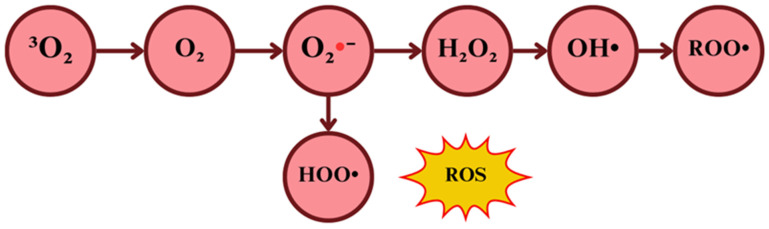
Reactive oxygen species transformation from triplet oxygen (^3^O_2_), singlet oxygen (atmospheric) (^1^O_2_), superoxide anion (O_2_•−), hydroperoxyl radical (HOO•), hydrogen peroxide (H_2_O_2_), hydroxyl radical (HO•), and peroxyl radicals (ROO•). ROS: reactive oxygen species.

Oxygen stands by far as the most vital element, for it bears the basis of biological oxidations, where most of the available energy for aerobic organisms is produced [32]. Yet in excessive amounts, it rends toxic effects, at both cellular and systemic levels [33]. The well-known toxic effect of oxygen is attributed to its chemical structure. Molecular oxygen in its simplest form has a unique electronic configuration, which makes it a radical itself. When an electron is added to the primary structure, the superoxide anion (O_2_•−), also known as the primary ROS, is formed. O_2_•− will interact with other available molecules to generate secondary ROS [34]. Given that ROS are electrically charged, they seek chemical neutralization by reacting with other molecules, causing oxidation, either reversibly or irreversibly, intrinsically increasing the phosphorylation (activity) of various proteins kinases and reducing the phosphatase activity of other proteins; the overall effect appears to enhance neuronal responsivity to sensorial inputs, although, high sustained levels of ROS might excessively oxidize proteins and membrane lipids, causing an abnormal responsiveness of neurons to afferent inputs, inducing central sensitization and pain [35,36]. These deleterious effects occur due to the structural alteration of organic biomolecules, such as protein oxidation, DNA strand breakage through guanine residue oxidation, RNA oxidation, the oxidation of polyunsaturated fatty acids in bilipid membranes, mitochondrial depolarization causing, consequently, apoptosis, and oxidative stress [6,19,37]. Among the most relevant reactive species containing oxygen, exist the hydroxyl radical (HO•), superoxide radical anion (O_2_•−), hydroperoxyl radical (HOO•), and peroxyl radicals (ROO•), and the non-radicals hydrogen peroxide (H_2_O_2_) and singlet oxygen (^1^O_2_), which in a series of transformations, involving the gain or loss of electrons, lead to changes in the charge and reactivity of the molecules (Figure 1) [38,39].

#### 2.1.1. Singlet Oxygen

**Figure 2:**
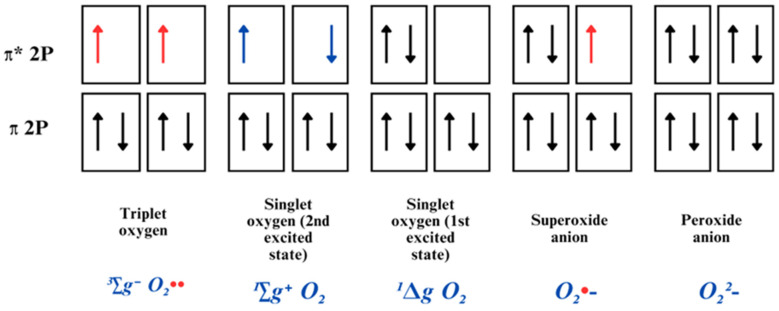
Outer valence and spin distribution of molecular oxygen in different electronic states. π*: antibonding orbital, π: bonding orbital, 2P: second level main energy of oxygen atomic orbital. Red arrows correspond to unpaired electrons with identical spin, blue arrows correspond to unpaired electrons with opposite spin, black arrows correspond to pair electrons.

O_2_ is considered a radical molecule due to the possession of two unpaired electrons with identical spin quantum numbers. The most stable form is triplet oxygen (^3^∑g^−^O_2_), also written ^3^O_2_, as commonly present in the atmosphere (Figure 2). It acts as a potent oxidant, and its spin arrangement enables it to readily interact with other molecules by accepting electrons individually, rather than releasing them. This specific characteristic clarifies why O_2_ tends to react more outstandingly with radicals than non-radicals [40].

The induction of an energy input can lead to the restructuration of electrons’ arrangement; once one of the unpaired electrons gains energy, the electron is excited and changes its spin, resulting in the creation of a more reactive dioxygen molecule, known as singlet oxygen [41,42]. This process eliminates unpaired electron spin restrictions, thereby enhancing its oxidative capacity, enabling interactions with proteins, DNA, and lipids [38].

#### 2.1.2. Superoxide Radical Anion

When an electron is added to the outer O_2_ orbital, containing an unpaired electron, a less radical molecule is formed, the superoxide radical anion (O_2_•−). This molecule possesses a single unpaired electron; therefore, it is less reactive than its preceding singlet oxygen [38]. O_2_•− is considered a “primary” ROS, produced during the ETC in mitochondria, via electron leakage towards molecular oxygen, in the process of oxidative phosphorylation which is known to be implicated in the pathophysiology of a myriad of diseases [43]. It can readily continue interacting with other molecules to produce “secondary” ROS, by means of direct interaction or via enzyme or metal-catalyzed processes [34,44].

In physiological conditions, most of the superoxide radical is present in its anionic form (O_2_•−), and only 0.6% is present in its protonated form, the hydroperoxyl radical (HOO•); nonetheless, the catalyzed reaction with HOO•− is a lot faster (k = 7 × 10^9^ M^−1^·S^−1^) than the uncatalyzed reaction involving two O_2_•− molecules (k < 0.3 M^−1^·S^−1^) to produce the dismutation of both radical forms, to H_2_O_2_ and O_2_, serving as a great source of H_2_O_2_ [45], which is of importance in the context of DNA damage, once O_2_•− does not react directly with DNA [44,46]. Much of the nuclear DNA damage is restored by the cell, yet, mitochondrial DNA (mtDNA) cannot be fixed in the same manner, which leads to accumulated damage over time, mitochondrial damage, and cell death [44].

Increased levels of O_2_•− have been largely observed in neuropathic pain conditions in dorsal horn neurons of rats’ spinal cords after nerve ligation [35,47], capsaicin-induced hyperalgesia [48,49], phenyl N-tert-butylnitrone (PBN) and 4-hydroxy-2,2,6,6-tetramethylpiperidine 1-oxyl (TEMPOL) [49,50,51], and intraplantar injection of O_2_•− [51], and have been found to mediate the development and maintenance of central and peripheral sensitization, either increasing nociceptive neurons’ excitability, activating calcium/calmodulin-dependent kinase II in glutamatergic spinal neurons, which leads to the presynaptic inhibition of GABAergic interneurons, causing disinhibition and enhancing neuroexcitability in pain signaling pathways and possible excitotoxicity [52,53].

#### 2.1.3. Hydrogen Peroxide

H_2_O_2_ is a very unstable and slowly decomposing molecule. It comprises an uncharged oxygen–oxygen (O-O) bond peroxide, with a pKa ± 10.8 at neutral pH, making it able to cross through cellular membranes. It has a high oxidizing power, yet it holds a rather slow rate of reaction with surrounding molecules; such characteristics enable it to exert its radical properties distant from the place it was formed, and confer an elevated capability to accumulate in cells, in relatively excessive concentrations [54].

The main source of H_2_O_2_ is formed by the dismutation of O_2_•− through the SOD-catalyzed reaction. Also, the NADPH-oxidase system is responsible for H_2_O_2_ production, since it performs the catalysis of NAPDH oxidation by O_2_, which yields NAD^+^ and H_2_O_2_ [29], as well as other enzymatic sources, such as glucose oxidase, amino acid oxidase, urate oxidase, and others [46].

Given that H_2_O_2_ is a by-product of oxidative stress in cell metabolism, for a long time it was considered a strictly harmful and undesired molecule, and it was not until a few decades ago that its role in several biological processes, such as cell differentiation, proliferation, inflammation, wound healing, and more, proved its value as a key redox signaling molecule [55,56]. Additionally, H_2_O_2_ plays a pivotal role in growth factor-induced signal transduction, thiol redox homeostasis, and mitochondrial function [46].

The physiological or pathological role of the molecule will drastically depend upon the cellular type, subcellular compartment, specific metabolic context, rate of production, and clearance. At micromolar concentrations it appears reactive, whilst at high concentrations it can trigger serious damage, by inactivating the glycolytic enzyme glyceraldehyde-3-phosphate dehydrogenase [54,57]. H_2_O_2_ is capable of directly oxidizing a significant number of molecules and inactivating enzymes containing thiol groups (RSH) or methionine (Met) residues in their active sites [58]. Moreover, H_2_O_2_ reacts efficiently with many other radicals, markedly NO, inducing the formation of peroxynitrite (ONOO^−^) [19].

H_2_O_2_ can increase the action potential frequency and amplitude in neurons of the dorsal root ganglion (DRG) in neuropathic models [59] and in neurons from within the subnucleus caudalis of the spinal trigeminal nucleus, causing facial pain induced by formalin injection in the lips of rats [60]. Additionally, H_2_O_2_ can trigger cGMP-dependent kinase Iα (cGKI), causing an increased neurotransmitter release from sensory neurons in the dorsal horn of the spinal cord of neuropathic rats [61], thus, contributing to glutamate homeostasis disturbance, which can potentially cause exacerbated synaptic conductance and amplified Ca^+^ influx in neuronal terminals, through the phosphorylation of subunit receptors of N-methyl-D-aspartate (NMDA), inhibition of glutamate transporter (GLT-1), and glutamine synthetase, instigating an advance of excitotoxicity, key in the development of neuropathic pain [62].

After the SOD-catalyzed dismutation reaction of H_2_O_2_, and under unhinged redox conditions, unbound metal ions, i.e., Fe^3+^, can catalyze the heterolytic cleavage of H_2_O_2_, resulting in the formation of hydroxyl radicals (OH•) and anion hydroxide (OH^−^), through a well-known reaction referred to as the Fenton reaction (k = 7.6 × 10^1^ M^−1^·S^−1^) [34,63].

#### 2.1.4. Hydroxyl Radical

The hydroxyl radical (OH•) is one of the most reactive radicals in biological systems, with a lifespan of 10^−8^–10^−9^ s. It can be produced by means of redox metal-catalyzed decomposition of H_2_O_2_, in the presence of transition metal ions, especially when these metal ions are iron or copper [64,65].

OH• may interact with other surrounding molecules by the abstraction of H^+^, transfer of electrons, or addition reactions, generating other radicals, although less reactive. Due to this high reactivity, OH• affects molecules’ non-selectively, by extracting carbon-bound H^+^ on relatively simple molecules, whether abundant or not, by attacking lipidic membranes which triggers oxidizing radical reactions, and by addition to nitrogenous bases, such as the deoxyribosyl backbone of DNA, which leads to oxidation, damaged bases, or strand break [44,66]. A direct link was established between the hydroxyl radical and DNA strand damage to neurons in the context of neuropathic pain induced by chronic constriction injury (CCI) in rats, where an increase in cellular dysfunction and apoptosis in neuropathic animals was observed and promptly reduced in animals treated with an aqueous extract of *Luehea divaricate* containing ROS scavengers. DNA strand break causes an activation of pain pathways that are also directly activated by hydroxyl radicals, and that can further release proinflammatory cytokines, increasing pain sensitization [67]. The hydroxyl radical is relevant for the attenuation of long-term potentiation and facilitation of long-term depression in GABAergic inhibitory interneurons, primarily contributing to pain through GABAergic disinhibition in the dorsal horn leading to enhanced excitatory transmission, hence resulting in neuropathic pain [68].

### 2.2. Reactive Nitrogen Species: Shemistry and Sources

#### Nitric Oxide and Peroxynitrite

Nitric oxide (NO•) is endogenously synthetized through a five-electron oxidative reaction involving L-arginine metabolization to L-citrulline, by specific nitric oxide synthases (NOSs). NO• has a role in a variety of physiological processes related to neurotransmission, synaptic plasticity in the CNS, smooth muscle relaxation, vascular tone, platelet aggregation inhibition, leukocyte adhesion inhibition, and antimicrobial immune response [69,70]. Due to its hydrophobicity, NO• can freely migrate through the cytoplasm and cellular membranes, and react distantly from its site of formation, and has a long lifespan (1–10 s) compared to other molecules [71].

Four main isoforms of NOSs have been recognized and located: a mitochondrial NOS (mtNOS) [69]; a neuronal NOS (nNOS), expressed in the postsynaptic terminal of neurons and Schwann cells; an endothelial NOS (eNOS), highly expressed in DRG consistently with allodynia, constitutively expressed and regulated by Ca^2+^/Calmodulin interaction; and an inducible NOS (iNOS), induced as a response to inflammation, trauma, or infection, which is not regulated by Ca^2+^, and highly expressed in the cytosol of glial cells [72,73,74]. The NO• produced by each isoform will perform different and specific functions, depending on the place where they are produced, e.g., the regulation of mitochondrial oxygen consumption and transmembrane potential via a reversible reaction with cytochrome c oxidase (mtNOS), communication among neurons (nNOS), the relaxation of blood vessels and maintenance of blood pressure in endothelium (eNOS), and contribution to defensive mechanisms of macrophages (iNOS) [73].

In the context of oxidative stress, the cytotoxic potential of NO• is due to the secondary production of oxidants derived from NO•−, which are by far more reactive, rather than the oxidative potential itself [75]. NO_2_• can interact with NO• to yield dinitrogen trioxide (N_2_O_3_), which is even more reactive than the priors, with a high ability to cause protein nitrosation and deamination reactions [76]. Therefore, NO• oxidizing potential is mainly related to its ability to interact with O_2_•− once they react to producing significant amounts of a much more oxidative molecule, the peroxynitrite anion (ONOO^−^) [77].

**Figure 3:**
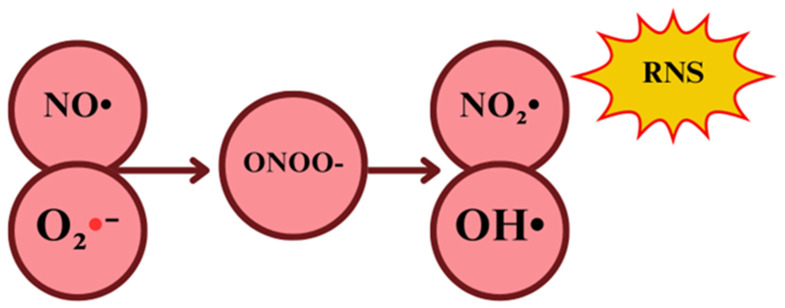
Peroxynitrite (ONOO^−^) formation from nitric oxide (NO•) and anion superoxide O_2_•−), and subsequent formation of hydroxyl radical (OH•) and nitrogen dioxide (NO_2_•) from peroxynitrite. RNS: reactive nitrogen species.

Regarding ONOO^−^, it is a short-lived (10 ms) one- or two-electron oxidant radical, produced by the interaction of oxygen and NO•, serving as the main NO•-derived oxidant. This process occurs in a competitive environment of O_2_•− against the enzymatic SOD-catalyzed dismutation to H_2_O_2_, NO• migration across the cell, NO• preferential reaction with guanylate cyclase, and finally, the reaction of O_2_•− with NO•, to ultimately yield ONOO^−^ in a potentially faster reaction when NO• concentrations increase (Figure 3) [76].

The ratio of the competitive environment determines the switch from physiological functions to pathological pathways signaling, once ONOO^−^ can act either as a cytotoxic effector molecule against pathogens or as an endogenous cytotoxic molecule towards host cells, based on the kinetics setting [78].

ONOO^−^ is able to engage with biomolecules like DNA, proteins, and lipids that lead to structural modifications in biomolecules, resulting in dysfunction such as interference with signaling pathways through protein amino acid nitration. The two major direct ways of ONOO^−^ acting on biomolecules are through one-electron (with transition metals) or two-electron (with thiols and CO_2_) oxidation reactions in an aqueous milieu [79].

On the other hand, ONOO^−^ can indirectly induce oxidation or nitration in amino acids including cysteine (Cys), tyrosine (Tyr), and methionine (Met) [76], as well as freely diffuse though lipidic membranes (ONOOH), and react via its secondary radicals with proteins and lipids in the hydrophobic milieu, thus, negatively impacting protein structural and functional roles, or inducing lipid peroxidation [80].

Both ONOO^−^ and NO• play significant roles in the settlement and perpetuation of neuropathic pain, through several molecular mechanisms, as described above; nevertheless, they can specifically contribute to the process through neuroexcitability enhancement of nociceptive neurons, activating Calcium/calmodulin-dependent protein kinase II (CaMKII) in excitatory glutamatergic neurons, potentiating synaptic transmission, increasing calcium influx, or causing GABAergic interneurons inhibition, leading to neuronal disinhibition [62,81]. Moreover, both species can cause transient receptor potencial (TRP) channel activation [82], critical for pain processing, preceding membranal protein and lipid nitrosative modifications, and Toll-like receptors (TLR) activation (iNOS) [37], consequently inducing nociceptive hypersensitivity. Mitochondrial stress is a major contributor to neuropathic pain conditions, and it has been seen that elevated levels of NOS- and NOX-derived species can cause mitochondrial homeostasis disturbance, and neuronal degeneration (BENNETT, 2014). As an overall effect, both species contribute to central and peripheral sensitization following nerve injury.

### 2.3. Endogenous Sources of Reactive Oxygen and Nitrogen Species (ROS/RNS)

**Figure 4:**
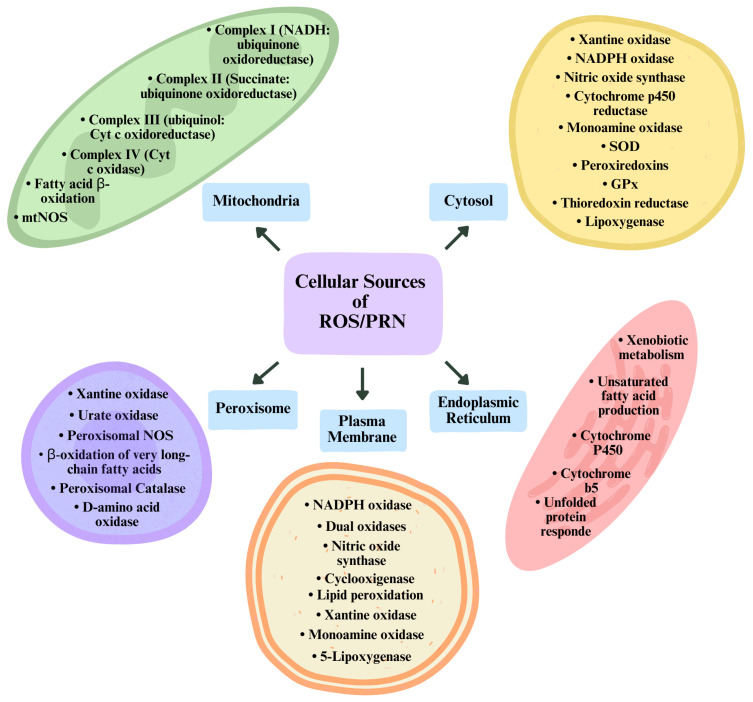
Endogenous sources of reactive oxygen and nitrogen species. Figure illustrates the major cellular organelles and enzymatic sources involved in the endogenous production of reactive oxygen and nitrogen species (ROS/RNS) during physiological cell functioning.

Several sources of ROS and RNS have been identified in neurons during normal and neuropathic pain processing, giving its fundamental role in signaling pathway modulation, discussed later in this paper. Although, as previously stated, ROS and RNS are exogenously and endogenously formed, the focus will be on the endogenous sources. Thus, at the cellular level, the majority of them come from enzymatic activity (Figure 4) [76].

#### 2.3.1. Endoplasmic Reticulum

The endoplasmic reticulum (ER) is formed as an intricately linked net with numerous tubules and sacs tightly interconnected and is thought to occupy around 10% of the cellular space and has a pivotal role in lipid and protein biosynthesis [83]. More specifically, it is involved in biosynthesis, folding, transport, and posttranslational modifications of proteins, whether of the plasma membrane, Golgi apparatus, or lysosomes, as well as calcium storage, and drug detoxification, in certain cell types [72,84,85].

In the smooth ER, reactive species are produced by any of two systems in charge of xenobiotic metabolism and unsaturated fatty acid production. Regarding xenobiotic metabolism, through a two-phase process, an initial monooxygenase reaction (phase I) using an NADPH-cytochrome P450 reductase and a complex of cytochrome P450 adds a polar group (OH^−^) in a lipophilic substrate, using O_2_ as a co-substrate, typically replacing a hydrogen atom, which introduces polarity into the molecule, and increases its water solubility. Following oxidation, the xenobiotic with added polarity undergoes a conjugation reaction (phase II) with endogenous molecules (glucuronic acid, sulfate, or glutathione), increasing further the hydrophilicity of the conjugate, facilitating its elimination [72].

In relation to the unsaturated fatty acid production, reactive species are produced by two mechanisms of action of cytochrome b5, which acts in electron transfer. First, cytochrome b5 can function as part of a multienzyme complex consisting of cytochrome b5, NADH cytochrome b5 reductase, and a specific desaturase. In this process, cytochrome b5 transfers electrons from NADH cytochrome b5 reductase to the desaturase. With the aid of molecular oxygen (O_2_), the desaturase inserts carbon-carbon double bonds into fatty acids, producing two molecules of water (H_2_O) [86]. Secondly, it can act through a direct electron transfer from the NADH cytochrome b5 reductase to the catalytic site of a fused cytochrome b5/desaturase, adding the same carbon-carbon bond, previously mentioned. In both cases, cytochrome b5 acts positively over the cytochrome P450 monooxygenase reaction, contributing to the availability of the second of two needed electrons for O_2_ to activate cytochrome P450, accelerating the catalytic process and increasing the formation of H_2_O_2_ and O•−, via the leakage of electrons to O_2_ by NADH cytochrome b5 reductase [72,87].

Altogether, and both present in the ER, cytochrome P450 and cytochrome b5 can contribute to reactive species production through diverse mechanisms. Given that cytochrome P450 enzymes participate in the metabolism of xenobiotics and endogenous molecules, some of the reactions involved occur with electron transfer to O_2,_ which subsequently contributes to ROS production. Moreover, cytochrome P450 enzymes suffer redox-cycling processes; in such processes, electron transfer between heme-iron centers and additional molecules occurs, which can result in ROS generation, more precisely O•^−^ and H_2_O_2_, as derivatives [88,89,90].

Into the bargain, it is believed that redox homeostasis in the ER can induce ER-associated stress, which triggers redox signaling mediators involved in ROS formation [46]. As an example, unfolded or misfolded proteins that accumulate in the ER lumen can elicit such a response, associated with chronic pain of neuropathic nature [91] As the first of two possible mechanisms, ROS can be formed during electron transfer from protein thiols to O_2_ by the Endoplasmic Reticulum Oxidoreductin-1/Protein Disulfide Isomerase (ERO-1/PDI). Next, during the protein misfolding process due to the depletion of glutathione reductase (GSH), ROS are formed; when GSH is used, thiols are restored and can readily interact with ERO-1/PDI, so as to be re-oxidized. In this cyclic and repetitive process, disulfide bond formation and scission generate ROS as derivatives [91,92,93].

Since ER stress in increasingly recognized as a relevant component in the development of neuropathic pain, ROS produced in the process play a significant role in it. Under high protein traffic in ER stress conditions, great quantities of misfolded proteins and ROS can be generated, triggering the unfolded protein response (UPR), which might lead to deviant cellular signaling, neurotoxicity, neuroinflammation, the disruption of cellular functioning, and apoptosis, particularly relevant in neuropathic conditions, where ROS levels are boosted [94,95].

ER stress and pain behavior were normalized in neuropathic animals undergoing nerve ligation, and BiP and *XBP1* expression in the spinal cord were reduced after treatment with tauroursodeoxycholic acid, an ER chaperone that aids the ER in protein folding, indicating the participation of ER stress in the development of neuropathic pain [96].

#### 2.3.2. Mitochondria

Among the most important functions of mitochondria are the production of energy in the form of ATP, either by the tricarboxylic acid cycle or oxidative phosphorylation, intracellular calcium concentration balance, and β-oxidation [72]. Almost all of these cellular functions are associated with reactive species production, and the mitochondria stands as one of the most important sources of reactive species with oxidant potentials. This amount of reactive species contributes to cellular damage and, at the same time, accounts for relevant redox signaling pathways [31].

It is widely accepted that oxidative phosphorylation and the ETC promote mitochondrial ROS formation in higher concentrations than any other mitochondrial activity; even so, the relevance of the other sites should not be disregarded, solely based on the percentage of production, given that there exist at least eleven different sites associated with substrate oxidation and oxidative phosphorylation electron leakage to O_2_ that also produce O• and H_2_O_2_ [97]. In general terms, if electrons leak individually or in pairs, they will generate O•− or H_2_O_2_, respectively. If they are transferred four-at-a-time, as expected, they will form H_2_O by inflowing to oxidative phosphorylation [97].

The mitochondrial ETC is responsible for ATP formation, through the process of oxidative phosphorylation. The oxidation of metabolites contained in energy sources undergoes a transfer of electrons to electron carriers, such as nicotinamide adenine dinucleotide (NAD^+^) and flavin adenine dinucleotide (FAD); the reduced form of these carriers transfers the electrons to the respiratory chain, and finally to oxygen [46,98]. During this process, as electrons pass through the mitochondrial complexes, some may leak out and react with O_2_, which then suffers either a reduction mediated by NADPH and xanthine oxidase, yielding O•− or by ubiquinol, a reduced form of ubiquinone [99].

Yet, it is notable that the production of O•− and H_2_O_2_ in the mitochondria will drastically depend on the protonmotive force, the NADH/NAD^+^ and the CoQH_2_/CoQ ratios, and the local concentration of molecular oxygen, which are unsteady and difficult to measure in in vivo biological systems. Thus, O•− and H_2_O_2_ levels will raise or lower depending on the redox state and the specific location of the electron donor: if they act upstream, causing an oxidation of the site, they will lead to reduced electron leak; if they act downstream, causing a reduction of the site, this will lead to increased electron leak. Altogether, this makes it troublesome to assume that any mitochondrial dysfunction will necessarily lead to increased O•− and H_2_O_2_ levels, or to oxidative stress; instead, a wider frame must be taken into consideration [97,100].

ROS levels are found to be increased in neuropathic pain, more specifically in microglia, astrocytes, and neurons of the dorsal horn in the spinal cord [17,48]. In the event of nerve injury, mitochondria dysfunction emerges as a consequence of excessive reactive species production, Exceeded by endogenous antioxidant systems, triggering mitochondrial fission and an unpaired expression of mitochondrial transcription factor A (TFAM) and mitofusin 2 (MFN2) proteins, responsible for mitochondrial biogenesis and fusion, respectively, and hence, contributing to oxidative stress and increased mitochondrial damage [101]. These events seem to be significantly regulated by the cellular energy sensor (adenosine 5′-monophosphate (AMP)-activated protein kinase) AMPK, which acts upon oxidative stress conditions, triggering peroxisome proliferator-activated receptor-gamma coactivator-1alpha (PGC-1α), which promotes mitochondrial gene expression related to biogenesis and mDNA replication and transcription, ultimately restoring mitochondrial function [102]. Furthermore, excessive ROS produced by mitochondria can trigger mitochondrial damage and exacerbate neuropathic pain through TRP channel activation, NMDA receptor upregulation, and GABAergic gating mechanisms inhibition, inducing NLRP3 inflammasome proinflammatory responses, and causing oxidative damage to neurons [50,103,104].

#### 2.3.3. Plasma Membrane

Given its direct contact with the extracellular space, a high exposure to oxidizing agents and reactions are plausible, leading to reactive species production [72]. Several metabolic processes can be disturbed due to oxidatively damaged lipids of the plasma membranes, which will precede transmembrane ion gradient alterations and the interruption of secretory and signaling functions [105].

The main source of reactive species generation is owed to the effect of NOX, which will indorse O•− production. NOX are a group of enzymes bounded to the plasma membrane present in a myriad of cell types [44]. NOX are constituted by six subunits: one Rho-GTPase, either Rac1 or Rac2, and five phagocytic oxidases (phox): gp91_phox_, p22_phox_, p40_phox_, p47_phox_, and p67_phox_. The first two are restricted to the membrane, and the last three are free in the cytoplasm [20]. Upon stimulation, when proper conditions allow the cytoplasmic subunit to migrate to the membrane subunits, all the components bound together, leading to allosteric modification and subsequent NOX activation. Once activated, NOX will catalyze the oxidation of cytoplasmic NADPH into NAD^+^, releasing an electron to O_2_, to produce O_2_•− onto the plasma membrane or in its outer side [106,107].

Out of the NOX family, NOX1, NOX2, and NOX4 have been linked to the neuroinflammation process and neuropathic pain development [53,108,109]. NOX1 is expressed in DRG, spinal cord neurons, and glial cells [110], and its inhibition by ML171 significantly reduced pain behavior through TRPA1-mediated ROS production in a model of neuropathic pain induced by peripheral nerve injury in rats [111]. NOX2 is expressed in microglia from the spinal cord, and in macrophages and neurons in DRG in neuropathic pain models induced by nerve injury; thus, its suppression was associated with decreased ROS production, microglia activation, and TNF-α and IL-1β, therefore, improving thermal and mechanical hypersensitivity [112,113]. Finally, NOX4 was detected in dorsal horn neurons and interneurons of the spinal cord, as well as in the injured nerve and DRG in peripheral nerve injury and diabetic neuropathy [114,115], contributing to ROS production, peripheral myelin integrity impairment through myelin protein zero (MP) and peripheral myelin protein 22 (PMP22) degradation, associated with apoptosis, and consequently, the exacerbation of the manifestations of neuropathic pain, all of which were reduced when NOX4 activity was inhibited in knock-out mice [116].

### 2.4. ROS/RNS-Mediated Cell Signaling

RONS play pivotal roles in cell functioning, by eliciting or regulating changes in signaling cellular messages, enzymatic activity, response to growth factor, the induction of inflammatory response [117], immune response, cellular adhesion, differentiation, proliferation, autophagy and apoptosis [118], gene transcription [119], and protein and membrane integrity [19,120], to name a few.

Given that the cellular activity is modified as a response to stimuli, mainly through the activation of gene transcription, the two recognized ways by which ROS participate in such activation, are by (1) the direct modification of transcription factors, which will be addressed to particular DNA promoters of target genes, or (2) via the activation of mitogen-activated protein kinase (MAPK) pathways, like extracellular signal-regulated kinases (ERK), c-Jun N-terminal kinases (JNK), and p38, which will lead to the phosphorylation and activation of other transcription factors, such as activator protein 1 (AP-1) and cAMP response element-binding protein (CREB) [121,122]. The two mechanisms by which cell signaling happen, by means of ROS, can be explained as (1) modifications of target protein molecules, once reactive species oxidize main Cys residues, or by (2) changes in intracellular redox state by the redox-buffering capacity of the cells [72]. The molecular alteration suffered by the former mechanism acts over enzymes, transporters, receptors, transcription factor regulatory sites, and allosteric and macromolecular sites [123], and can alter their localization, activity, and DNA-binding ability of the transcription factors, thus repressing or enhancing the transcription of target genes [120,124].

Although some reactive species can cause irreversible structural modifications over molecules, with low specificity (ex.: OH•), permanently damaging them, other moderate oxidants, such as H_2_O_2_, will primarily target Cys residues of proteins, more precisely thiol groups (-SH) [72]. Thiol groups from Cys residues can undergo several oxidative modifications, and the process typically occurs as follows: initially, ROS oxidize the -SH group of Cys to form sulfenic acid (-SOH); sulfenic acid (-SOH) can further react with a neighbor -SH group, either from the same protein or from another protein, yielding the formation of a disulfide bond (-S-S-). Under certain conditions, such as excessive reactive species, sulfenic acid (-SOH) can be further oxidized to sulfinic acid (-SO_2_H) and then to sulfonic acid (-SO_3_H). These two last higher oxidation states are usually irreversible. On the other hand, even though sulfenic acid formation has been seen as damaging, it has its own relevance as a redox-sensing signal for cellular functions, due to the reversible oxidation by ROS [125,126].

Other targets of redox signaling exist, such as the iron–sulfur (Fe–S) cluster or Tyr residues, but the list could be vastly extensive for the purpose of this paper. Therefore, this section takes into consideration those redox-signaling targets deemed more sensitive to nitroxidative effects and/or implicated in the regulation of protein function, associated with transcription factor control and well-characterized mechanisms, and that contribute to the regulation of oxidative stress, offering insights to how cells sense and respond to oxidative stress and neuropathic pain.

#### 2.4.1. NRF2–ARE Pathway

**Figure 5:**
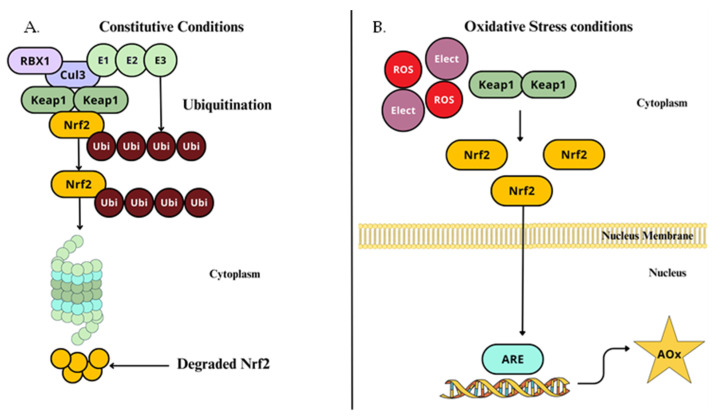
NRF2–ARE pathway. (**A**). Constitutive conditions of normal Nrf2 degradation by ubiquitination process in the cytoplasm. (**B**). Oxidative stress conditions of Nrf2 release by KEAP1 and translocation to the nucleus towards DNA regions responsible for the ARE encoding. AOx: antioxidant; Elect: electrolytic stressors; Ubi: ubiquitin.

Nuclear factor erythroid 2-related factor 2 (NRF2) is a redox-sensitive leucine zipper (bZIP) transcription factor [127] that has a significant role in neuropathic pain modulation through the inflammatory response and oxidative stress management by hyperalgesia and allodynia regulation, regulating the expression of HO-1 and IL-10 [127,128]. It is expressed in microglia in the spinal cord, DRG, and sciatic nerve [129]. NRF2 binds to specific DNA sequences to control transcription from DNA to RNA [130]. It is activated in response to oxidative and electrophilic stress [131]. Its major function is to initiate the expression of a wide range of cytoprotective genes once it has a role in maintaining redox homeostasis [56]. In quiescent conditions, in the cytoplasm, NRF2 is bound to the Kelch-like ECH-associated protein 1 (KEAP1) dimer, which acts as an adaptor protein that links NRF2 to the Cullin 3 (Cul3)-based E3 ubiquitin ligase complex, which causes its ubiquitination, by attaching to lysine residues on NRF2, through a process involving ubiquitin-activating enzymes 1 (E1), ubiquitin-conjugating enzymes (E2), and E3 [132]; this tags NRF2 for subsequent degradation by the 26S proteosome into small peptides and this interaction maintains low NRF2 cellular levels, as expected in normal conditions (Figure 5A) [133].

When an increase in oxidative stress occurs, NRF2 is released from KEAP1, accumulates in the cytoplasm, translocate to the nucleus, and heterodimerizes with specific regions of DNA encoding antioxidant response elements (ARE), detoxification, and cytoprotective proteins, such as heme-oxygenase 1 (HO-1) [134], CAT, SOD, and others (Figure 5B) [130,135].

As previously stated, KEAP1 is found to have a double function, as an oxidative and electrophilic stress sensor, and negative regulator of NRF2, by degradation via ubiquitination [132]. Out of 25 cysteine residues of KEAP1, three are among the most critical for redox sensing, responsible for NRF2 dissociation (Cys^151^) and degradation (Cys^273^ and Cys^288^) [136]. KEAP1 is highly reactive and able to undergo modifications in response to alterations in the inner cellular redox state; these Cys can be oxidized or covalently modified, inducing the formation of disulfide bonds [137].

Thus, conformational variations reduce the affinity for NRF2, ultimately preventing KEAP1 from enabling the ubiquitination and degradation of NRF2. This allows NRF2 to stabilize, and move forward with its transcriptional inducing capacity, which then triggers cellular protective antioxidant [138], anti-neurotoxic [139], neuroprotection [140], and anti-inflammatory responses [141].

Decreased NRF2 and HO-1 levels were observed in neuropathic rats after vincristine administration, where oxidative stress, DNA damage, neuronal cell damage, and inflammation, evidenced by increased 8OH-dG, diminished glial fibrillary acidic protein (GFAP), and nuclear factor kappa-B (NF-κB) were present, and promptly reversed after quercetin administration, showing the role played by the NRF2 pathway, and the protective effect of quercetin against neuropathic pain caused by vincristine [128]. To evaluate the role of NRF2 in neuropathic pain, NRF2 siRNA was administered to CCI-induced rats, which prominently decreased the expression of *NRF2* RNA and NRF2 and HO-1 protein and worsened the hyperalgesia behavior (withdrawal threshold and latency) [127]. Also, a study investigating the involvement of NRF2 expression in spared nerve injury in rats with our without an anhedonia phenotype, classified after a hierarchical cluster analysis of a sucrose preference test, showed that NRF2 expression was significantly decreased in the medial prefrontal cortex, hippocampus, and spinal cord of anhedonian rats, compared to non-anhedonian ones. After the NRF2 activator sulforanphane was administered, a reduction in mechanical withdrawal threshold was observed, while sucrose preference remain unchanged, due to NRF2 level normalization in the animals with the anhedonia phenotype [142].

#### 2.4.2. NF-κB Signaling Pathway

The nuclear factor κ-light-chain-enhancer of activated B cells (NF-κB) is a pleiotropic inducible transcription factor implicated in pro-survival regulatory inflammation and immune functions, cellular adhesion, differentiation, and cell proliferation. It is activated in a wide variety of neuroinflammation-associated stimuli via pro-apoptotic pathways [143,144]. It is composed by homo- and heterodimers of five structural protein members, of which Rel-A(p65), Rel-B, and c-Rel contain C-terminal transactivation domains (TADs), and p50/p105 and p52/p100, which also serve inhibitory functions [120].

Conversely, in the nervous system, it is composed mainly by p50/Rel-A heterodimers [145], but the most widely studied dimers are those of Rel-A and p50, also referred to as NF-κB_can_, once they participate in the canonical activation pathway. On the other hand, Rel-B and p52 form a dimer referred to as NF-κB_non_ for they participate in the non-canonical activation pathway, discussed later [146]. Additionally, NF-κB contains Rel-1-homology domains (RHD) that bear a nuclear localization signal, accountable for dimerization, detection, and binding to DNA, as well as communication with the NF-κB inhibitor (IκB) [147].

**Figure 6:**
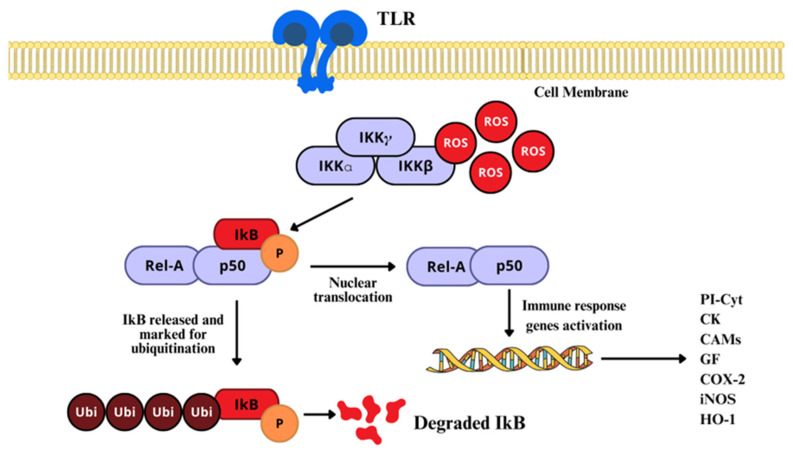
Canonical pathway of NF-κB activation via TLR (Toll-like receptors) and IKK complex oxidation/nitration leads to IκB–NF-κB complex separation and leads to NF-κB nuclear translocation. PI-Cyt: pro-inflammatory cytokines; CK: chemokines; CAMs: cell adhesion molecules; GF: growth factors; COX-2: Cyclooxygenase-2; iNOS: Inducible Nitric oOxide Synthase; HO-1: Heme-Oxygenase-1.

As previously stated, the NF-κB pathway is mainly activated by either of two kinase-dependent pathways, the canonical and the non-canonical. However, in the nervous system, the most studied and well known is the canonical pathway, which includes a series of cytokines and Toll-like receptors (TLR) [144]. In normal conditions, NF-κB dimers form complexes with IκB, to retain them in the cytosol, by masking their nuclear localization signal (NSL), and also preventing NF-κB from binding to DNA by masking its DNA-binding sites [147]. There, H_2_O_2_, HOCl, _1_O^2^, and peroxynitrite act via the oxidation or nitration of specific critical Cys residues on the IκB–kinase (IKK) complex, which terminates the joining of IκB to NF-κB. Once IκB is structurally modified, activated NF-κB is released and translocated to the nucleus (Figure 6) [19,148].

**Figure 7:**
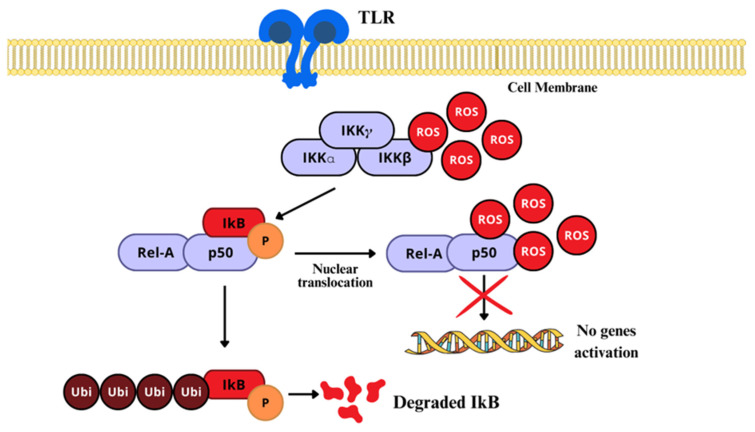
NF-κB pathway inactivation via reactive oxygen and nitrogen species direct interaction with the DNA-binding region’s Cys leads to inhibited gene activation in the nucleus under increased levels of ROS/RNS.

When phosphorylated at a specific regulatory aminoacidic residue by the IKK complex, IκB is ubiquitinated and marked for 26S proteosome degradation, still in the cytosol [149]; NF-κB is freed for nuclear translocation, to activate the transcription of target genes [46], where it binds to enhancer regions of specific genes, such as proinflammatory cytokines, chemokines, adhesion molecules, growing factors (GF), and promoters of COX-2, iNOS, and heme-oxygenase [149,150]. On the other side, H_2_O_2_ can regulate NF-κB by direct interaction with the Cys of the DNA-binding region (Figure 7) [151]. In that sense, higher than normal levels of RONS in the nucleus can prevent NF-κB from interacting with DNA, reducing its transcriptional activity, although increased H_2_O_2_ can induce peroxiredoxin 1 activity, which is a H_2_O_2_ scavenger.

A diverse array of genes with sometimes differing functions respond to NF-κB_can_, i.e., pro-survival genes such as inhibitors of apoptosis (IAP), *Bcl-2*, *Bcl-x*, *Bcl-w*, *Bfl-1*, *BIRCS*, and *MnSod*; yet NF-κB_can_ can also regulate pro-apoptotic genes like *p53*, *TNF-α*, *Bax*, *c-myc*, *APO-1*, and *FasL* [146]. Given the extremely specialized types and subtypes of neuronal cells, it is a major challenge to study NF-κB, due to traditional techniques limitations in their ability to mirror realistic and accurate locations and activities of proteins that are regulated by specific dimer combinations and several post-translational modifications.

NF-κB is widely implicated in spinal microglial activation to M1 phenotype in the establishment of neuropathic pain, as seen in the examination of growth and differentiation factor 11 (GDF11), an inhibitor of macrophage activation, and its impact on microglia polarization, thermal and mechanical hyperalgesia in sciatic nerve injury in mice, showing a marked reduction of pain behaviors, enhancing the switch from M1 to M2 phenotype through the modulation of the transforming growth factor beta receptor type 1 (TGF-βR1)/SMAD2/NF-κB pathway [152,153]. Another study aimed to investigate the participation of Pellino 1 (Peli1), a component of the E3 ubiquitin ligase, in the activation of microglia in neuropathic pain induced by sciatic nerve injury. It was found that higher levels of Peli1 and NF-κB expression, as well as pain behavior after spared nerve injury (SNI), induced neuropathic pain. Also, the administration of intrathecal Peli1 shRNA prior to SNI and siRNA after the establishment of SNI, reduced the expression of Peli1 and NF-κB in the spinal dorsal horn of mice, alleviating the thermal and mechanical threshold through NF-κB pathway modulation, as Peli1 is needed for the ubiquitination of proteins regulating NF-κB activation [154].

#### 2.4.3. MAPK/AP-1 Signaling Pathway

The activator protein 1 (AP-1) is a heterogenous family constituted by the basic-region leucine zipper (bZIP) transcription factor, accountable for the regulation of cellular responses to numerous extracellular stimuli, including ROS [155]. This vast group is represented by the members of the Jun (c-Jun, JunB, and JunD), Fos (c-Fos, FosB, Fra-1, and Fra-2), Maf (c-Maf, MafA, MafB, MafG/F/K, and Nrl) and ATF (ATF2, ATF3, B-ATF, JDP1, and JDP2) protein subfamilies [156,157]. They harbor a C-terminal leucine zipper domain that allows them to form hetero- and homodimers, which subsequently bind to specific DNA sequences known as 12-O-tetradecanoylphorbol-13-acetate (TPA)-responsive elements (TRE) or cAMP response elements (CRE) in the promoter regions of target genes, where they regulate gene expression, mediated by basic N-acid-rich domains, parallel to the leucine zipper [157]. The specific arrangement of the dimers composing the AP-1 complex establishes the interaction patterns, molecular affinities, and functional specificity that represent their tasks in the cell [158,159].

**Figure 8:**
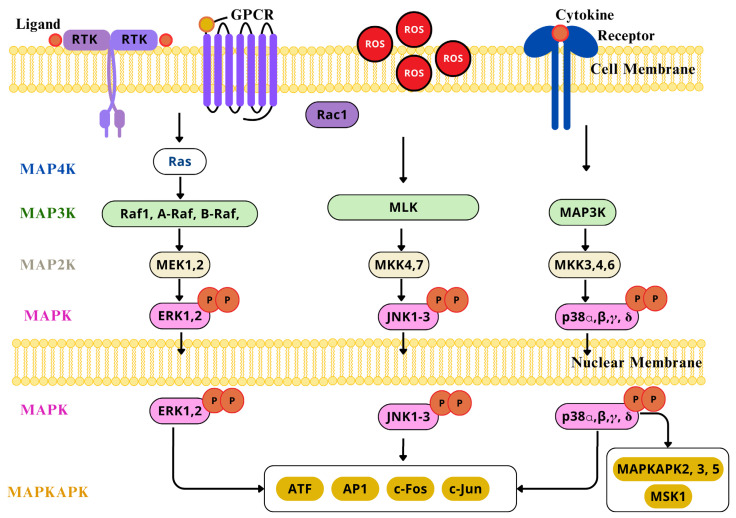
MAPK/AP-1 signaling pathway. MAPKs are regulated by a cascade of phosphorylation and dephosphorylation of threonine and serine residues, triggered by tyrosine kinase receptors (RTK) activation, protein tyrosine kinase (TK), cytokine receptors (CR), heterotrimeric G protein-coupled receptors (GPCR), growth factors (GF), or ROS/RNS. ATF: activating transcription factor; AP-1: Activator Protein—1; MLK: Mixed-Lineage Kinase; MEK: MAPK/ERK Kinase; MKK: Mitogen-Activated Protein Kinase; ERK: Extracellular Signal-Regulated Kinase; JNK: c Jun N-terminal Kinase.

AP-1 transcription factor can be redox activated by ROS, ROS-dependent factors, cytokines, GF, neurotransmitters, UV light, and ionizing radiation [160]. The increased exposure to H_2_O_2_, OH•, and O•− can directly determine the redox state of several conserved cysteine residues included in the AP-1 pathway [161,162]. Also, AP-1 can be indirectly regulated by ROS signaling pathways, like NF-κB and interferon-γ. The AP-1 transcription factor family is a target of the ROS-sensitive MAPK cascade for phosphorylation, this leads to enhanced transcriptional activation [163,164]. ROS activate ERK1/2, JNK, and p38 MAPKs (Figure 8), which are crucial mediators for signal transduction pathways leading to AP-1 activation, through the phosphorylation of c-Jun on serine and threonine residues, enhancing its stabilization and c-Fos transcriptional activity over genes involved in neuron, microglia, and astrocyte activation, in a sequential manner [165], particularly relevant in dorsal horn neurons of the spinal cord and DRG, and playing distinct roles in different cells across the development of neuropathic pain [166]. The accurate redox control of AP-1 expression and activation is essential for homeostasis, and its alteration has been implicated in altered neurons states, for it is a key molecular switch that controls the expression of downstream proinflammatory factors [167]. Nevertheless, the regulation of AP-1 activity is complex as it depends on severa factors such as, changes in *Jun* and *Fos* gene transcription and mRNA expressionthe effects on Jun and Fos protein turnover, post-translational modifications of Jun and Fos proteins that modulate its transactivation capacity, and finally, on the interaction with other transcription factors that can positively or negatively affect AP-1 activity [168].

An oxidative shift of the intracellular thiol/disulfide redox state, or ROS, are accountable for significant activation of the MAPK cascade elements, as seen in neurons [169,170]. They consist in a series of protein kinases that activate each other in specific sequences. MAPKs are regulated by the phosphorylation and dephosphorylation of threonine and serine residues, and are triggered by tyrosine kinase receptor activation, protein tyrosine kinases, cytokine receptors, heterotrimeric G protein-coupled receptors, and GF [39]. The major MAPK cascades include ERK1/2 (extracellular signal-regulated kinase 1 and 2), JNK (c Jun N-terminal kinase), the p38 pathway, and ERK5, each of which has specific upstream activators, downstream targets, and biological actions [171].

Each cascade is composed by three central kinases (MAP3K, MAP2K, and MAPK); an additional upstream MAP4K and downstream MAPKAPK components are included. Respectively, each cascade has its own signal transmitted by the successive phosphorylation and activation of sequential kinases, that at a given point lead to the phosphorylation of the target regulatory proteins by the MAPK and MAPKAPK constituents [172]. With the aim of executing their functions, MAPKS and MAPKAPKs phosphorylate and regulate their substrates, so as to induce and regulate the de novo gene expression of other transcription factors and suppressors, and chromatin remodeling proteins, at the nucleus level, after being physically transported across the nuclear membrane [173].

The ERK1/2 cascade is activated through various membrane receptors, but mostly via GTPase Ras, which receives extracellular signals at the plasma membrane, that in turn conscript the Raf-1 and B-Raf (MAP3K) tier of the cascade to the plasma membrane, probably through homo- or hetero-dimerization and phosphorylation [174]. Therefore, the kinases activating the Raf components are considered MAP4Ks. Afterward, the signal is transmitted to the MEK1/2 (MAP2K), through the phosphorylation of serine residues in their activation loop. The activated MEK1/2 transmit the signal to ERK1/2 (MAPK) also by the phosphorylation of the threonine and tyrosine regulatory residues in the Thr-Glu-Tyr domain, in the activation loop [175]. Ultimately, the signals pass on to the MAPKAPK elements, RSKs, MNKs, and MSKs, and other substrates located in the cytoplasm or the nucleus. Both ERK1/2 and MAPKAPK can phosphorylate substrates involved in cellular proliferation, differentiation, neuroplasticity, stress response, pro-survival, and pro-apoptotic processes [176]. As a matter of fact, ERK is phosphorylated in dorsal horn neurons of the spinal cord, in DRG neurons, and in peripheral nerve terminals after nerve injury, contributing to peripheral and central sensitization [165,177]. ERK activation occurs in a sequential manner, starting in the dorsal horn neurons after spinal nerve ligation (SNL) within 10 min through the transcriptional modulation of NK-1 and prodynorphin [178,179], followed by microglia for some days, having its most robust expression in 3 days, and finally, in astrocytes for weeks, supporting the role of ERK in astrocytes in the maintenance of neuropathic pain, whose inhibition reduced behavior associated with neuropathic pain [165]. Isoliquiritigenin reduced mechanical and thermal hyperalgesia by a CCI in mice, inhibiting the activation of spinal microglia and neuroinflammation through the ERK pathway inhibition [180].

The JNK cascade is usually activated by stress signals such as UV radiation and cytokines [181]. It includes MLK (MAP3K), MKK4/7 (MAP2K), and JNK (MAPK). Once activated by stimuli dependent and independent of stress and mitogens, it transmits their signals to GTPases, such as Rac1, that activate the MAP3K-level kinases directly or via MAP4Ks [182]. The MAP3K tier transmits the signals through the phosphorylation of the threonine and serine residues in the activation loop, which consequently activates the MKK4/7; finally, these kinases activate any of the components of the MAPK level by the direct phosphorylation of tyrosine and threonine residues in the activation loop. As expected, the JNKs and their respective MAPKAPKs phosphorylate substrates in the cytoplasm and nucleus that modulate the transcription of genes mediating apoptosis, immunological responses, and neuronal activity [183]. JNK-1 is activated in early stages (three days) and late stages (21 days) in GFAP- expressing astrocytes in neuropathic pain caused by SNL, and transiently (12 h) in DRG neurons after SNL, specifically in small C-fibers; therefore, this supports the idea that spinal astrocytes contribute to the continuance of neuropathic pain [166]. The neuroprotective effect of Orexin B was evaluated in a model of neuropathic pain induced by CCI in rats. Orexin was found to be effective in improving mechanical and thermal hyperalgesia, preventing microglia activation reducing Iba1 levels and preventing JNK/NF-κB signaling pathway activation [184].

As for the p38 cascade, it is activated by stress signals and environmental stressors, but it also responds to normal cellular processes, like inflammatory cytokines [185]. It involves MKK3/4/6 (MAPKK) and p38 (MAPK) [172]. After activation by stress conditions or receptors, the signals are transmitted via GTPases, MAP4K, and MAP3K, in a similar manner to the JNK cascade. The MAP3K level of the cascade phosphorylates and activates MKK3/6, and under certain conditions, MKK4 (MAP2K) components of the p38 cascade [186]. At this point, the p38 isoforms and functional unconventional spliced forms at the MAPK level are activated via the phosphorylation of the threonine and tyrosine residues in the regulatory domain in their activation loop. Lastly, the signals are transmitted by the p38, or MAPKAPKs to target motifs accountable for regulatory processes [172]. p38 MAPKs are particularly expressed in the superficial laminae of dorsal horn neurons in the spinal cord, whose increase correlates with lower mechanical withdrawal and thermal latency thresholds in SNL-induced neuropathic pain [166]. Increasing evidence points to the role of p38 MAPK in the activation of spinal microglia after nerve injury and its contribution to neuropathic pain, via downstream mechanisms of proinflammatory mediators after nuclear translocation, where it influences the gene expression of TNF-α, IL-1β, and IL-6, critical mediators of neuroinflammation and pain that also exacerbate other pain signaling pathways [187]. p38 MAPK involvement in the pathophysiology of neuropathic pain mediating microglial activation was evaluated in SNL rat models treated with electroacupuncture to assess the analgesic effect of such treatment; it was found that a significant decrease in p38 MAPK activation and enhanced mechanical withdrawal threshold, both in the ipsilateral and contralateral dorsal horn of the spinal cord, not only demonstrated the beneficial effect of electroacupuncture, but the potential of targeting the p38 MAPK pathway to treat neuropathic pain [188].

#### 2.4.4. Phosphoinositide 3-Kinase (PI3K)/Akt Pathway

The PI3K pathway constitutes a conserved family of kinases present in the inner side of the plasma membrane, capable of acting specifically on the D-3 position of the inositol ring of inositol phospholipids [189]. Such phosphorylation produces phosphatidylinositol-3,4,5-triphosphate (PIP3), phosphatidylinositol-3,4-biphosphate (PIP2), and phosphatidylinositol-3-phosphate (PIP) [190]. The PI3K enzymes, when activated, transmit intracellular signaling cascades concerning signal transduction, protein synthesis, vesicular traffic, cytoskeletal reorganization, cell growth, proliferation, survival, metabolism, apoptosis, autophagy [189,191], drug resistance in response to GF, such as epidermal growth factor (EGF), platelet-derived growth factor (PDGF), nerve growth factor, (NGF), vascular endothelial growth factor (VEGF), and brain-derived neurotrophic factor (BDNF) [122,192]. PI3K has also been found to be essential in the initiation and maintenance of neuropathic pain [193].

Although in mammalians cells, the PI3Ks exists in three classes (I, II, and III), and each of these have different structural characteristics, functional homologies, and substrate specificity, the most prevalent and significant in the nervous systems is the class I PI3Ks, where they play a crucial role in neuronal development, synaptic plasticity, and neuroprotection [194]. Class I PI3K are further divided into class IA and class IB subgroups [191,195,196]. The heterodimeric class IA PI3Ks are composed of a regulatory subunit p85, including five polypeptides p85α, p85β, p55α, p50α, and p55γ, and a catalytic subunit p110 (p110α, p110β, or p110δ), which are activated by receptor tyrosine kinase (RTK) and G-protein-coupled receptors (GPCRs), small G protein Ras, and cytokines [197]. The p110α and p110β are ubiquitously expressed in all cell types, while p110δ expression is more restricted to leukocytes [198]. The catalytic p110 subunit contains a C-2 region for membrane anchoring; a Ras-binding region (RBD); an adaptor-binding region (ABD), which holds an N-terminal region for interaction with the regulatory p85 subunit; a helical region; and a catalytic region. The p85 regulatory subunits have in common a p110 binding region (inter-Src-homology-2 region, iSH2) and two SH2 regions for p110 ABD binding, constitutively [189,199]. On the other hand, class IB PI3Ks enzymes are formed by the p110γ polypeptide, associated with an adaptor subunit p101 or p84 and p87, to form heterodimers, p101/p110γ, or p84/p110γ, which are primarily activated by GPCRs, due to the interaction of its regulatory subunit with the subunit of trimeric G protein [200]; this isoform is present in the nervous system.

**Figure 9:**
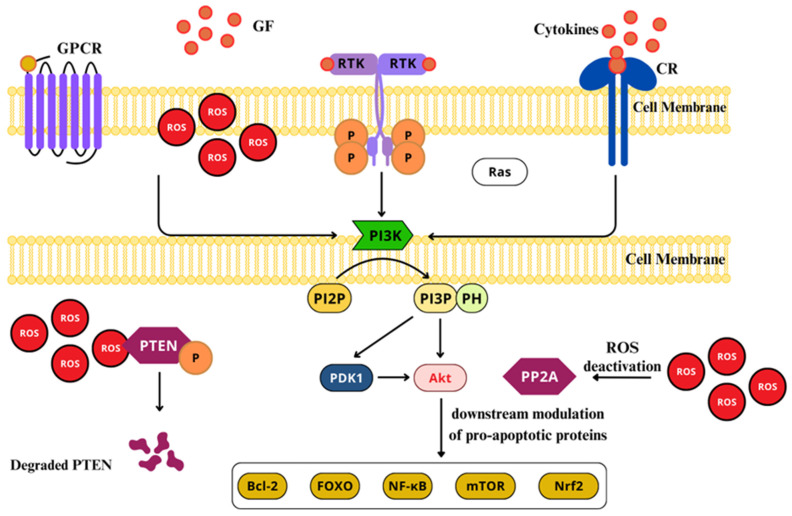
Phosphoinositide 3-kinase (PI3K)/Akt pathway. PI3K is activated by RTKs or GPCRs, GF, CR, and ROS. Once activated, PI3K transforms the PIP2 to PIP3. This serves as a second messenger for Akt (protein serine/threonine kinase) and phosphoinositide dependent kinase 1 (PDK1) activation. Akt activation allows downstream modulation of pro-apoptotic proteins such as Bcl-2, caspase 9, fork head transcription factors (FOXOs), NF-κB, and mTOR. ROS can inhibit phosphatase and tensin homolog (PTEN), which negatively regulates PIP3 synthesis and inhibits Akt activation, leading to further activation of the PI3K/Akt signaling pathway. Phosphatase 2A (PP2A), a Akt/PKB inhibitor, can be deactivated by ROS.

Either of these PI3Ks catalyzes the phosphorylation of PIP2 to produce PIP3 [201,202,203]. Typically, its activation starts with the union of a ligand to RTKs or GPCRs, through its regulatory p85 subunit. In non-activated states, the p85 subunit remains bonded to the catalytic subunit p110 ABD region, through its iSH2 region, which stabilizes the p110 catalytic subunit. Once properly activated through cytokines, GF, insulin, and LPS, the SH2 regions bind to their respective receptors or adapter proteins, leading to the allosteric activation of the p110 catalytic subunit of the PI3K [194,204]. When activated, PI3K transforms the PIP2 to PIP3, which serves as a membrane-embedded second messenger, inducing the repositioning and activity of pleckstrin-homology (PH) region-containing proteins, in the inner side of the plasma membrane [192]. Among these proteins, can be included Akt (protein serine/threonine kinase), also knowns as PKB, and phosphoinositide dependent kinase 1 (PDK1) [202]. Akt activation permits the transportation on protein kinase to the cytoplasm and nucleus, where it downstream modulates pro-apoptotic proteins, such as Bcl-2, caspase 9, fork head transcription factors (FOXOs), NF-κB, mTOR, and glycogen synthase kinase 3 (GSK3) (Figure 9) [192,197,205].

ROS and RNS interact with specific amino acids residues on tyrosine phosphatases, protein tyrosine kinase, and protein kinase C, that can trigger kinase cascades, MAPKs, and PI3Ks [206]. ROS can directly activate PI3K, increasing its downstream signaling pathway [207]. At the same time, H_2_O_2_ can oxidize and inhibit phosphatase and tensin homolog (PTEN), which negatively regulates PIP3 synthesis and inhibits Akt activation, hence, leading to a further activation of PI3K/Akt signaling pathway [208]. Additionally, ROS enhance the phosphorylation of PTEN through casein kinase II, allowing PTEN to undergo proteolytic degradation [209]. Moreover, phosphatase 2A (PP2A), an Akt/PKB inhibitor, can be deactivated by ROS [209,210]. Nevertheless, low ROS levels cause the oxidation of Akt/PKB -S-S- bridges, promoting PP2A association with the Akt/PKB complex, promoting the short-term activation of the Akt/PKB pathway [211,212].

The dysregulation of this pathway has major implications in neuropathic pain [193]. PI3K/Akt inhibition or alteration leads to elevated states of ROS production, membrane depolarization, mitochondrial membrane stability impairment, the diminishment of oxidative phosphorylation, and ATP production [213,214]. The class I PI3K catalytic subunit p110α has a role in controlling ROS levels at a physiologic rate through the NRF2/ARE-dependent pathway [215], which, if not regulated, could lead to activation of the autophagy machinery [206]. The dysregulation of the PI3K/Akt pathway has a relevant role in the establishment of chronic pain conditions derived from traumatic lesions [192,193,216,217]. PI3K has also been linked to TRK systems NGF/TrkA and BDNF/TrkB activation, displaying central sensitization and hyperalgesia mediation induced by G-CSF [218], and mediating pain induced by plantar incision in mice [216]. Additionally, PI3K mediates peripheral and central sensitization and hyperalgesia caused by the intraplantar injection of EphrinB1-Fc, triggering the EphrinBs (ligand) and EphBs (receptor) pathway activation [219], affecting neuronal excitability, leading to an increased firing of pain signals, contributing to pain hypersensitivity [220]. PI3K was proved to be crucial for the DRG and dorsal horn neurons’ upregulation of the kinesis family member 1a (Kif1a), following a positive correlation between increased levels of PI3K/Akt/CREB, and Kif1a protein and mRNA expression in a chronic pain model induced by chronic constriction injury. The process starts with CREB phosphorylation by PI3K, which recruits the DNA demethylation TET1 towards the *Kif1a* promoter region, inducing an upregulation of its expression, which then leads to pain sensation. Such insight offers a novel target for managing neuropathic pain, since the inhibition of PI3K led to decreased Kif1a expression, and alleviating pain-associated behaviors [221].

#### 2.4.5. Calcium Signaling

**Figure 10:**
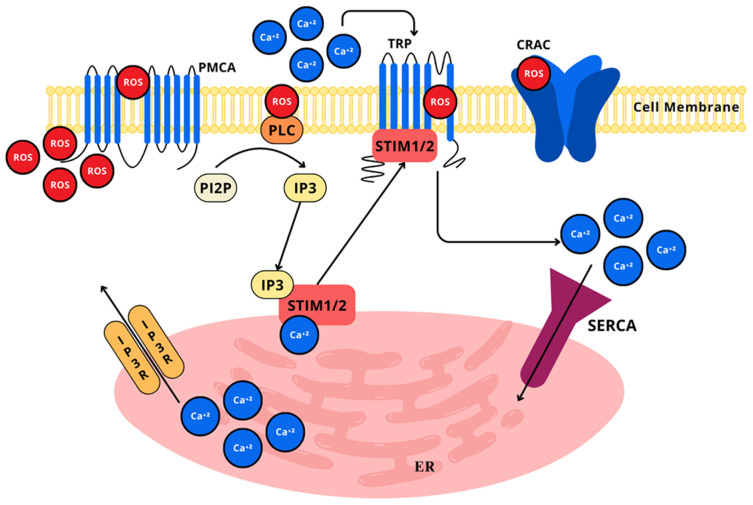
Calcium signaling induced by reactive species. ROS can stimulate calcium signaling in cells of the nervous system, initiating lipid peroxidation and activation of phospholipase C (PLC) and inositol 1,4,5—triphosphate (IP3) receptor (IP3R)-mediated calcium signaling. Also, ROS/RNS can directly oxidize or nitrosylate cysteine residues in Ca^2+^ channels and can influence Ca^2+^ channel gene expression, affecting their quantity and proportion in the cellular membrane, open-time probability, and trafficking. PMCA: plasma membrane calcium ATPase; STIM1/2: Stromal Interaction Molecule 1/2; CRAC: calcium release-activated calcium channel; SERCA: endoplasmic reticulum calcium ATPase.

Divalent calcium ions (Ca^2+^) are widely implicated in a plethora of pro-survival and pro-apoptotic cell processes, considering that it is one of the most relevant second messengers for intracellular signaling in most cells, but particularly in neurons and astrocytes, where it serves intricate and integrated functions like dendritic response to neurotransmitters, gene expression through nucleus signaling, and neurotransmitter release from presynaptic terminals [222]. Ca^2+^ aids in communicating depolarization status and synaptic activity to neurons [223]. The biochemically elegant mechanism through which it exerts signaling properties relies on the calcium concentration gradient relation of the extracellular space and the cytosolic space, as well as the relation of the cytosolic space and intracellular compartments, such as the endoplasmic reticulum and mitochondria [223]. In this context, given that Ca^2+^ is a main element for maintaining and controlling neuronal excitability, it is not absurd to consider the high energetic demand that this implies for neurons, as all of the Ca^2+^ that enters the cytosolic space must be removed from within, through plasma membrane calcium ATPase (PMCA), sodium-calcium exchanger (NCX), mitochondrial calcium uniporter (MCU), mitochondrial efflux systems, endoplasmic reticulum calcium ATPase (SERCA), calcium release channels, and cation-dependent calcium pumps (Figure 10) [224].

In the mitochondria, Ca^2+^ is transported through the MCU, against the concentration gradient, by means of electrochemical gradient–mitochondrial membrane potential; once in the mitochondria, Ca^2+^ forms inactive precipitates bound to inorganic phosphates [225]. When Ca^2+^ is not bound, it is transported back to the cytosol by the NCX. Nevertheless, when mitochondrial levels of Ca^2+^ are excessively high, this will force its way out through the mitochondrial permeability transition pores (mPTP); this marks the initial steps towards the release of pro-apoptotic proteins and the beginning of cell death through necrosis or apoptosis [226,227]. Cytosolic Ca^2+^ level increases mediated by ROS induce the AP-1 proteins c-Fos and c-Jun [39,228], as well as PKC-α activation through oxidative stress [229].

As formerly stated, the mitochondria is a paramount producer of reactive species, through the ETC as by-products, mainly O•− and H_2_O_2_, as well as through the TCA cycle, α-ketoglutarate dehydrogenase, or pyruvate dehydrogenase complexes, which mainly produce O•− and H_2_O_2_, or through enzymes not related to the ATP production, yet residents of the mitochondrial membrane, such as monoamine oxidase (MAO), cytochrome b5 reductase (Cb5R), and glycerol–3–phosphate dehydrogenase [230]. Altogether, the mitochondria can be considered a producer and a target itself of ROS actions, due to its intricate interaction, mutual regulation, and complementary functions, either in physiological or pathological conditions [231].

ROS can stimulate calcium signaling in cells of the nervous system, as seen in cases where astrocytic MAO produced H_2_O_2_, initiating lipid peroxidation and the activation of phospholipase C and inositol 1,4,5—triphosphate (IP3) receptor (IP3R)-mediated calcium signaling [232]. Astrocytes in hypoxia conditions have been shown to trigger Ca^2+^ signaling as a response to ROS; also, RONS can directly oxidize or nitrosylate cysteine residues in Ca^2+^ channels, specifically in the pore-forming α-1-subunit [233], altering its conformational structure and function, modifying neurotransmitter release and synaptic plasticity, essential for learning, memory, and chronic pain [234]. Additionally, RONS can influence Ca^2+^ channels’ gene expression, affecting their quantity and proportion in the cellular membrane, open-time probability, and trafficking [235]. Two major types of channel proteins seem to be involved in receptor-induced Ca^2+^ signals, the store-operated Ca^2+^ channels (SOC) and transient receptor potential channels (TRP). Both are of major importance for cell functions and thus have been accounted for in the entrance and release of Ca^2+^ through plasma membrane channels, and from its sites of storage, respectively. TRP, which are known to transport mainly Ca^2+^ ions, have six non-voltage sensitive transmembrane channels, divided based on their activation mechanism and N-termini or C-termini regulatory domain, the canonical (TRC1–TRC7), melastatin-related TRP (TRPM1–TRPM8), and vanilloid receptor-related TRP (TRPV1–TRPV4) [233].

All of the TRP family members are redox sensitive and participate in redox regulation and oxidative stress, either through activation by H_2_O_2_ mediated by ADP-ribose or cyclic ADP-ribose reacting in the binding cleft of the C-terminal [236], by other oxidizing agents that sensitize TRP to pH alterations [237], or by the modulation of SOC, which is regulated by the translocation of the ER Ca^2+^ sensors stromal interaction molecule 1/2 (STIM1/2) to the plasma membrane, where they activate Orai channels to ignite calcium entry and storage [238]. The TRP channels are of paramount relevance in the processing and discrimination of heat, cold, pain, and stress components and in the establishment of neuropathic pain derived from chemotherapy treatment. Paclitaxel can cause severe peripheral neuropathic pain, demonstrated by a mechanical withdrawal threshold decrease in rats, which was inhibited after intrathecal and intraperitoneal administration of the TRPV1 antagonist, capsazepine; TRPM8 protein expression was also reduced in DRG neurons. The topical application of menthol increased TRPM8 activity and reduced TRPV1 activity. In that sense, TRPV1 upregulation and TRPM8 inhibited activity are related to neuropathic pain induced by paclitaxel [239]. Following nerve injury or neuron dysfunction, TRP channel expression is altered affecting neuronal excitability, neuroplasticity, and neuroinflammation, leading to neuropathic pain, as evidenced by TRPC1, TRPC4, TRPC5 and TRPC6 increased expression associated to mechanical hypersensitivity in SNI [240,241,242].

In this sense, both mitochondrial ROS production and Ca^2+^ have physiologic roles in normal cell functioning. Nevertheless, dysfunction in mitochondrial ROS production and Ca^2+^ equilibriums also play a major role in the pathogenesis of chronic pain states and cell death, once neurons are vulnerable to redox and energetic disturbances [243].

#### 2.4.6. Unfolded Protein Response (UPR) Pathway

As formerly mentioned, when the ER undergoes stressing conditions, such as alterations in redox homeostasis, hypoxia, altered Ca^2+^ regulations, sustained hyperglycemia, and the accumulation of unfolded/misfolded proteins in the ER lumen [244,245], a series of adaptative/protective or maladaptive responses initiates, named the unfolded-protein response (UPR), which directly or indirectly affects the ER itself, or other organelles [92]. The primary objective of this elicited response is to reestablish normal ER function and homeostasis; nevertheless, when stress conditions are indeed critical and chronic, the cell is driven towards dysfunctionality, oxidative stress, ER stress, and ultimately, cell death [246]. ER stress is widely implicated in neuronal injury [247,248]. SNL induces an increased expression of ER stress markers such as *sXBP1* and BiP, as well as inducing the activation of the ATF6 and PERK/eiF2 pathway in dorsal horn neurons of the spinal cord [249]. The use of chaperones such as tauroursodeoxycholic acid has been effective in reversing ER stress, elicited by the UPR in neuropathic conditions like CCI, diabetic peripheral neuropathy, and that induced by formalin [248,249,250]. The influence of the UPR towards neuropathic pain involves central and peripheral sensitization, either by increased ER stress markers, or impairing calcium signaling, for the trigger of nociceptive pathways, leading to hyperalgesia.

**Figure 11:**
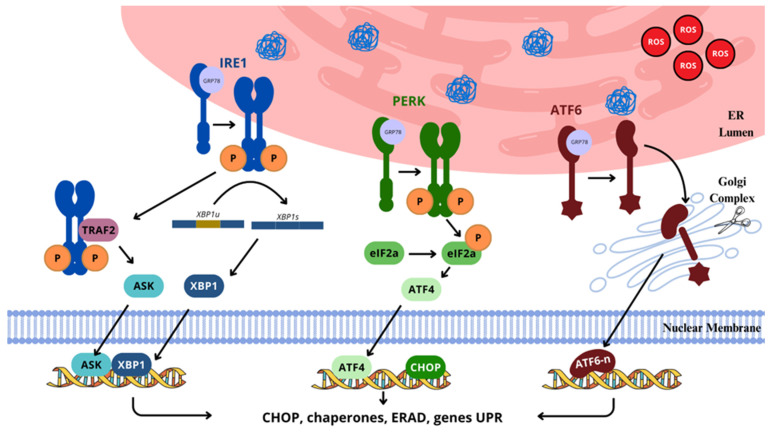
Unfolded protein response (UPR) pathway. Under non-stressed conditions, GRP78 attaches to unfolded protein response (UPS) sensors, IRE-1α, ATF-6, and PERK. In the presence of unfolded/misfolded proteins, GRP78 is detached from the UPR sensors; once activated, GRP78 induces complex pro-survival autophagy pathways, the antioxidant ERAD pathway, and ER regeneration, or pro-apoptotic and ferroptosis mechanisms. Also, ROS produced serves as a signal for activation or modification of the cellular stress response, once ROS-oxidated cysteine residues in the UPR sensors can mediate UPR signals. IRE-1α: Inositol-Requiring Kinase 1; ATF-6: Activating Transcription Factor 6; PERK: RNA-activated protein kinase (PKR)-like Endoplasmic Reticulum Kinase; eiF2a: Eukaryotic Initiation Factor 2 Alpha; XBP1: X-box Binding Protein 1; CHOP: C/EBP Homologous Protein; ASK1: Apoptosis Signal-regulating Kinase 1; TRAF2: Tumor Necrosis Factor Receptor-Associated Factor 2.

The initiation and development of the UPR involves three proximal ER stress-sensor transmembrane kinases/transcription factor, namely inositol-requiring kinase 1 (IRE-1α), activating transcription factor 6 (ATF-6), and double stranded RNA-activated protein kinase (PKR)-like endoplasmic reticulum kinase (PERK), each of which has its own ER branch, but also works complementarily to restore normal ER functions, which are, under non-stressed conditions, attached to the immunoglobulin heavy chain binding protein (GRP78/BIP) through their amino terminals at their ER luminal ends (Figure 11) [92,251]. The ER embodies a highly oxidizing-folding environment to enable disulfide bond formation as one of the fundamental steps in normal protein structural formation; thus, 25% of the ROS produced by cells comes from ER activity [245].

This oxidative process of intramolecular disulfide bond formation, known as oxidative protein folding (OPF) is one of the most usual posttranslational protein modifications [252]. The catalyzation of the disulfide bond formation is primarily carried out by the thioredoxin protein disulfide isomerase (PDI), through thiol-disulfide oxidation. PDI are formed by four thioredoxin (Trx) domains, namely a, a’, b, and b’, and a KDEL ER retention group of c-domain [253]. The redox state of the catalytic a-domains in the PDI enzyme establishes the oxidase or isomerase activity, depending on the CGHC (cysteine-glycine-histidine-cysteine) active site motifs; thus, PDI in its reduced state, due to the active site cysteines within the CGHC motif present as thiols (-SH), allows PDI to disrupt incorrect disulfide bonds, acting as disulfide reductase, as well as rearrange incorrect disulfide bonds into correct ones, acting as isomerase, while in the oxidized form, the active site cysteines within the CGHC are present as disulfide (-S-S-) bonds, allowing PDI to oxidize substrates, and to form new disulfide bonds between cystine residues, working as an electron acceptor [254].

On the other hand, b’ domains are responsible for the determination of misfolded or unfolded protein, through the identification of hydrophobic exposed terminals of the proteins [255]. H_2_O_2_, in addition to being the main source for oxidation, produced by O_2_, is also used by peroxidases to shape disulfide bonds to re-oxidize PDI. The cyclic reoxidation of PDI in the active sites help maintain the redox balance within the ER, which is more oxidative than the cytosolic environment. Yet, under highly oxidizing conditions, the CGHC motif can be hyperoxidized to its cysteine sulfinic acid or sulfonic acid form, turning it inactive, and unable to catalyze further reactions, leading to an accumulation of unfolded or misfolded proteins [254].

Successively, the ER membrane-associated oxidoreductin (ERO-1), that possesses two cysteine pairs on a flexible loop and on the CXXC motif, transfers sulfhydryl electrons from the reduced PDI to O_2_, yielding H_2_O_2_, which adds to maintaining the oxidizing environment [246,256]. In this sense, the ER redox state is tightly related to ER homeostasis, which depends on ROS production during disulfide bond formation in protein folding, to participate in the pro-oxidizing environment in the ER [257]. Additionally, quiescin sulfhydryl oxidase (QSOX), an FAD domain- and Trx domain-containing enzyme, catalyzes the formation of disulfide bonds, via free thiol group oxidation on cysteine residues of substrate proteins, transferring electrons from the cysteines of substrates to the cysteines in the Trx domain of QSOX, which are then shifted to its FAD domain. The reduced FADH_2_ domain acts as an electron acceptor, ready to be re-oxidized, conveying the electrons to O_2_, yielding H_2_O_2_, allowing QSOX to reenter a new cycle of disulfide bonds formation [258].

Further increase in ROS formation is triggered by Nox4 activity after UPR [259]. Additionally, another source of ER-stress is attributed to ROS-dependent Ca^2+^ depletion from the ER, which restrains the ability of Ca^2+^ to activate ER chaperones, such as calreticulin and calnexin. This can be explained due to the fact that IP3R, the ryanodine receptor (RyR), and Ca^2+^ pumps are redox regulated. For example, SERCA activity is inhibited when the Cys674 residue undergoes sulfoxidation and is solely activated after the same cysteine residue undergoes glutathionylation by NO; IP3R’s sensitivity to IP3 and RyR activity is enhanced by ROS, leading to augmented Ca^2+^ release from the ER [260].

However, excessive RONS can lead to the dysregulation of these receptors, contributing to ER and cellular stress and death through disrupted calcium homeostasis. As another source of ER oxidizing environment, when misfolded proteins appear, disulfide bonds are corrected by reduction through GSH, resulting in higher oxidized glutathione (GSSH) rates; this also participates in redox ER homeostasis [92].

In this process, paradoxically, the UPR induces increased ROS levels, while at the same time, ROS-mediated ER stress is expected to be restored by UPR. In this context, ROS ER levels increase due to higher ERO-1-PDI activity, but the ROS produced also serve as a signal for the activation or modification of the cellular stress response, once ROS-oxidated cysteine residues in the UPR sensors, IRE-1α, ATF-6, and PERK can mediate UPR signals, being activated to induce complex pro-survival autophagy, the antioxidant ERAD pathway, and ER regeneration, or pro-apoptotic and ferroptosis mechanisms [94]. Similar to ROS, RNS induce nitrosative stress, being linked to UPR-mediated cytotoxicity via cysteine residue inactivation in the active sites of PDI [261].

## 3. Antioxidant Enzymes

The nervous system exhibits a high oxidative metabolic activity rate with a consequent preeminent amount of reactive species production via enzymatic and non-enzymatic reactions as by-products of normal neuronal functioning [20], being particularly elevated during neuron and glia activation by glutamate and ATP [262]. Moreover, the relatively low levels of antioxidants [263], the high membrane surface to cytoplasm rate, and the high lipid content make neurons particularly vulnerable targets to oxidative damage over macromolecules and lipid peroxidation [264]. Although, as mentioned above, redox signaling works as an intrinsic sensor for oxidative stress, serving several physiological functions, and when the cellular antioxidant systems fail to prevent the overproduction of such reactive species, detrimental pathways can be activated, inducing cellular and tissue damage.

**Figure 12:**
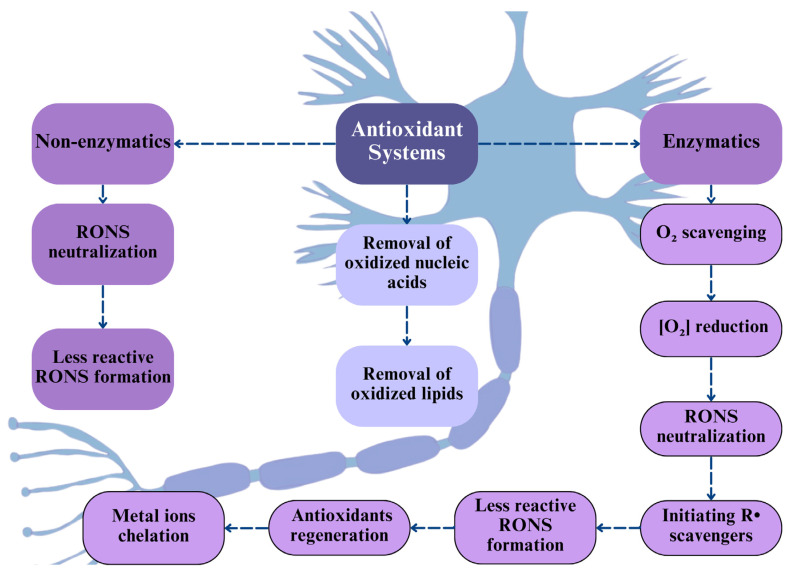
Enzymatic and non-enzymatic antioxidant endogenous systems’ mechanisms of action.

The cellular antioxidant systems, that is, those compounds present at a lower concentration in relation to oxidizable substrates that are able to hinder or preclude the oxidation of the substrate [265], are composed by enzymatic and non-enzymatic molecules and are chemically structured for and capable of reducing other compounds with an oxidizing potential of different chemical natures [65]. The first line of endogenous defense is constituted by an enzymatic antioxidant system that includes (superoxide dismutase) SOD, (catalase) CAT, and (glutathione peroxidase) GPx, whereas the second line of defense is formed by non-enzymatic antioxidants, such as antioxidant enzyme cofactors, reactive species scavengers, oxidative enzyme inhibitors, and Cu, Fe, Zn, and Mn transition metals, by participating in reactions that neutralize ROS directly or catalyzing the formation of less reactive species (Figure 12) [263,266]. Further processes of the restoration of damage by a repair system can be activated, eliminating oxidized nucleic acids by proteolytic enzymes, and lipids, either by phospholipases or peroxidases [16,267].

For the purpose of this review, the focus will be on preventative enzymatic antioxidants, as these are considered the most important among the endogenous antioxidant systems for their compelling contribution to the normal redox state, and for their role in promoting the synthesis or regeneration of further antioxidant or pro-survival mechanisms [46,268]. In addition to SOD, CAT, and GPx, which are considered to be the first front line of the endogenous antioxidant defense system, the Trx enzyme and thioredoxin reductase (TrxR) will also be included for their enzymatic nature, as opposed to those considered by other authors [269].

### 3.1. Superoxide Dismutase

The superoxide dismutase family (SODs) includes transition metal-containing enzymes present in all living organisms existing in the presence of oxygen. SODs are considered one of the most powerful antioxidant enzymes against biological oxidants and possess the ability to transform two O•− molecules into H_2_O_2_ and O_2_ in a pH-independent medium [268]. In mammals, three forms of SOD are acknowledged. The Cu, Zn-containing SOD-1 is a homodimer constituted by eight beta strands, with one Cu atom serving for catalytic function and one Zn atom relevant for structural stability, in each of the two subunits, respectively; it is ubiquitously expressed, and found predominantly in the cytoplasm, but also expressed in the mitochondrial intermembrane space and the nucleus [270,271]. SOD-2 is a homotetrameric enzyme that contains Mn in its active site. It is found in the mitochondrial matrix where the pH is considerably higher (pH approx. 7.8), compared to that of the intermembrane space (approx. 7.0–7.4); it is expressed at different amounts depending on the type of cell, being significantly higher in those cells that encompass elevated proportions of mitochondria [268].

**Figure 13:**
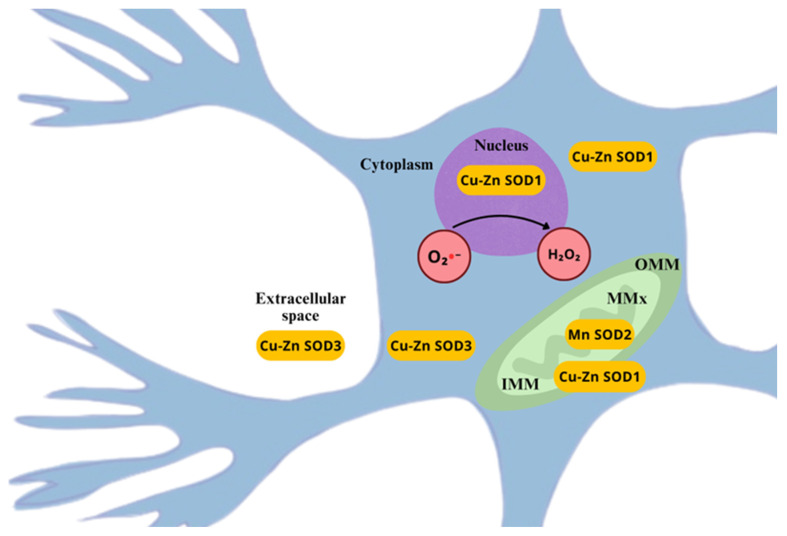
Superoxide dismutase (SOD) sites of expression in neuronal cell. IMM: internal mitochondrial membrane; OMM: outer mitochondrial membrane; MMx: mitochondrial matrix.

They perform their dismutation power over the O•− molecules produced in the ETC, providing electrons from the metal core directly to the negatively charged O•− molecules and a protonation to yield H_2_O_2_ [271]. Finally, the Cu- and Zn-containing enzyme SOD-3, produced intracellularly and found predominantly in the extracellular space [272], is homotetrameric in humans and mice, but dimeric in rats (Figure 13) [273]. As opposed to SOD-1 and SOD-2, which are ubiquitously expressed, SOD-3 is diversely expressed across different types of cells, and found to be highly expressed in heart, lungs, and pancreas, but low in the brain [273]. Additionally, SOD-3 is generally less responsive to direct induction by O•− molecules or other oxidants, due to its presence and role in the extracellular matrix, where exposure to regulatory signals other than those encountered in the intracellular space are found. SOD3 is regulated in conjunction with signaling molecules and cytokines, such as interleukin-1 (IL-1β) and TNF-α, as part of a coordinated response to inflammation, contributing to modulate oxidative stress in the extracellular environment [274].

The dismutation reaction catalyzed by SOD is considered to be highly efficient given that it happens at a diffusion-limited rate of ~2 × 10^9^ M^−1^·s^−1^, that is ~10^4^ the rate constant during spontaneous dismutation [275]. In the process, an electron reduction and protonation of the O•− radical results in the formation of H_2_O_2_, by means of an initial step of O•− radical binding to the core Cu(II) ion, resulting in the oxidative transfer of an electron to O•− and the production of reduced Cu(I); then, a second O•− radical is bound to a partly protonated Arg143 in the anion-binding site of the SOD that oxidizes the O•− radical. Moreover, His63 donates a proton and an electron from reduced Cu(I) to O•− radical, producing H_2_O_2_, and the oxidation of Cu(I) to Cu(II), to pursue further dismutation [276].

Changes in SOD expression or function are linked to chronic pain. Maropitant treatment increases SOD expression, reducing pain threshold in peripheral nerve injury, often marked by neuronal loss [248,271]. The enhanced expression of SOD-1 was observed to reduce lipopolysaccharide-mediated superoxide production, suppress cyclooxygenase (COX)-2 and iNOS expression, and confer protection against microglia-mediated inflammatory responses [277]. Compromised SOD-2 function is a common characteristic of pathogenesis associated with oxidative stress in brain injury [278]. Moreover, an increase in SOD-2 expression avoided neuronal death from oxidative stress [279].

### 3.2. Catalase

**Figure 14:**
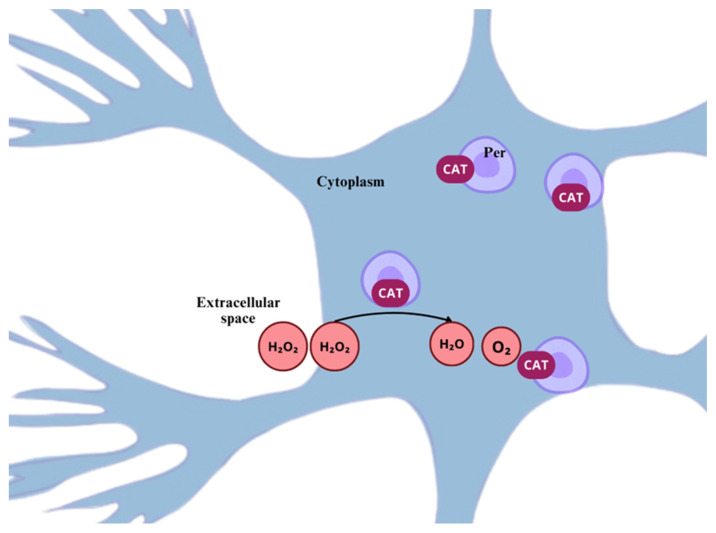
Catalase (CAT) sites of expression in neuronal cell. CAT is characterized by the ability to catabolize the rupture of two H_2_O_2_ molecules to form two molecules of H_2_O and one of O_2_. Per: peroxisome.

Catalase (CAT) is a tetrameric heme-containing enzyme, formed by four tetrahedrally arranged subunits of 60 kDa each, containing an active heme group and NADPH bound to each subunit [280]. It is part of the front-line defense antioxidant system, commonly found in mammalian cells as part of the peroxisome [263]. CAT is produced due to oxygen exposure and is characterized by the ability to catabolize the rupture of two H_2_O_2_ molecules to form two molecules of H_2_O and one of O_2_, through a two-step reaction, using Fe as a cofactor (Figure 14) [281]. It is expressed ubiquitously in central nervous system cells, including neurons and glia, where it protects cells by the detoxification of H_2_O_2_, allowing the body to tolerate and adapt to oxidative stress, as an adaptative response [282]. In addition to its protective effects against ROS, CAT offers influence over several pathways and metabolic processes such as growth, proliferation, and apoptosis [283] and influence over redox-sensitive signaling molecules, like protein kinases and transcription factors, through the modulation of H_2_O_2_ levels, impacting cellular responses to environmental or endogenous stressors [284].

The reaction is described as a two-step reaction, as follows: initially, a compound I, the covalent oxyferryl species Fe(IV) possessing a porphyrin π cation radical, is formed from Fe(III), via the reduction of one H_2_O_2_ molecule; following this, the Fe(IV) compound is decomposed through redox reactions via two-electron transfer from the second H_2_O_2_ molecule, which works as an electron donor to the reaction, therefore, producing O_2_ and H_2_O, and restoring the compound I to its resting state Fe(III) [285]. After this the final products, O_2_ and H_2_O, are released from the catalase, and the latter returns back to its original state, to pursue further reactions, binding and decomposing another H_2_O_2_ molecule. CAT is maintained chemically and structurally available after repeated cycles of chemical reductions of H_2_O_2_, contributing to steady O_2_ concentrations states, and redox balance [285].

Also, it is considered one of the most powerful antioxidant molecules in biological systems, as it has the highest turnover of all enzymes, degrading millions of H_2_O_2_ per second [268]. The inhibition of CAT activity induced increased cytotoxicity and higher ROS rates, denoting a pivotal role in oxidative balance [263].

Therefore, CAT dysfunction or deficiency is thought to cause impaired glucose metabolism and lipid accumulation [286], as well to contribute to oxidative stress and neuronal damage in paclitaxel-induced neuropathic pain, where it is decreased together with NRF2, SOD2, and HO-1, and can be reversed by cannabidiol (CBD) and tetrahydrocannabivarin (THCV) by the upregulation of catalase, but also other protective proteins such as p-AMPK [287], contributing to pain relief and neuronal protection.

CAT is increasingly recognized as a therapeutic target for diseases, founded on CAT-based therapeutic strategies providing neuroprotective effects through integrity and function neuronal preservation, for treating or averting neuroinflammatory events, using viral-cultured CAT or gene therapy, delivering *CAT* genes, and enhancing endogenous *CAT* gene expression. Ongoing innovative studies are utilizing protein engineering to enhance CAT-based therapies, aiming for improved stability, activity, and specificity, and demonstrating promising potential to expand therapeutic applications [288].

### 3.3. Glutathione Peroxidase

**Figure 15:**
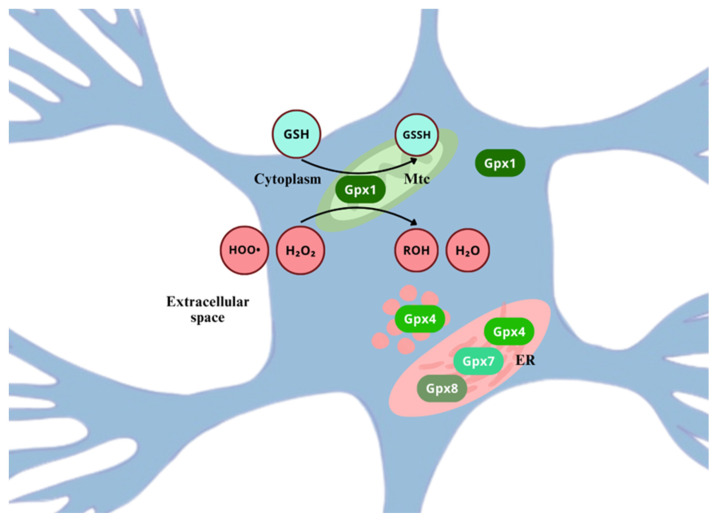
Glutathione peroxidase (Gpx) sites of expression in neuronal cell. Gpx drives the enzymatic reduction of H_2_O_2_ and hydroperoxides via oxidation of reduced glutathione (GSH) to its oxidized form (GSSH) in a ping-pong mechanism involving oxidation of the GPx followed by a two-step reduction.

The family of antioxidant enzymes glutathione peroxidase (GPx) consist of a group of eight isoforms (GPx1–GPx8) identified to date and present in all living organisms. Among them, GPx1 is the most abundantly found in almost all tissues and is highly expressed in the cytoplasm and mitochondria; GPx2 is found mostly in the gastrointestinal tract and human liver; GPx3 is found in the plasma and in the extracellular space; GPx4 is expressed ubiquitously, but especially in the brain, testis, and sperm cells where cell membranes need to be protected from lipid peroxidation; GPx5 is specific to the epididymis; GPx6 is expressed in the olfactory epithelium and embryonic tissues; and GPx7-8 are expressed predominantly in the ER, where they play a role in protection from oxidative stress and maintaining the oxidative balance for oxidative protein folding and the secretory pathway (Figure 15) [289]. Chemically, GPx are oligomeric enzymes, formed by subunits arranged with either a selenocysteine or cysteine amino acid redox-sensitive active site, both playing main functions in the catalytic reduction of hydroperoxides and H_2_O_2_, which are transformed to alcohols (ROHs) or H_2_O, respectively [290].

The GPx family has a vast number of physiological functions, although the most important role played is related to the enzymatic reduction of H_2_O_2_ and hydroperoxides via the oxidation of reduced glutathione (GSH) to its oxidized form (GSSH) in a ping-pong mechanism involving the oxidation of the GPx followed by a two-step reduction [291]. As a first phase, the catalytic site of GPx consisting of a selenocysteine or a cysteine amino acid is oxidized to selenenic acid or sulfenic acid, respectively, by hydroperoxides or H_2_O_2_ which successively are transformed to their corresponding reduced forms, alcohols (ROHs) or H_2_O [292]; thereafter, the second phase involves the return of GPx to its initial reduced state, through a series of reduction reactions by the GSH and NADPH as a cofactor. Here, the two-step reduction process starts with selenenic acid and sulfenic acid return to their oxidized states, selenocysteine and cysteine, with the help of two GSH molecules, which become oxidized (GSSG) in the process [289]. The return of the oxidized GSSG form to the reduced GSH form involves the enzyme glutathione reductase (GR); for such, NADPH binds to the active site of the GR that has an FAD cofactor. Here, the NADPH donates electrons to FAD, reducing it to FADH_2_, which in turn transfers these electrons to the disulfide bond in GSSG, disrupting the bond and yielding two molecules of GSH. As FAD is reduced, NADPH is oxidized to NADP+, which is released from the enzyme, finishing the cycle [289].

The intracellular depletion of GSH and decreased GPx4 activity caused by ferroptosis, an iron-dependent cell death pathway seen in pathological processes, results in the accumulation of lipid peroxides, the Fe(II)-catalyzed oxidation of lipids, and an increased production of reactive species [293]. In a spinal cord injury model in rats, GPx was upregulated following treatment with L-carnitine and atorvastatin, as opposed to non-treated animals, improving neuronal function [294]. Gpx is also upregulated in SNL- induced neuropathic pain [295]. Gpx4 was downregulated in a model of spared nerve injury in rats; after methyl ferulic acid administration, Gpx4 levels were increased, accompanied by a significant NOX4, ferroptosis- related protein ACSL4 decrease, and consequent mechanical withdrawal threshold and thermal withdrawal latency improvement [296]. Another study assessing whether sirtuin 2 (SIRT2) attains a neuroprotective effect in spared nerve injury-induced neuropathic pain, found enhanced levels of Gpx4 and ferroportin 1 (FPN1) after SIRT2 recombinant adenovirus intrathecal administration, reducing intracellular iron accumulation and oxidative stress, hence, attenuating mechanical hypersensitivity [297]. It has also been demonstrated that Gpx4 upregulation can reverse and inhibit ferroptosis-induced stress, blocking neurons and glia activation from the dorsal horn of spinal cord in neuropathic pain induced by chronic constriction injury in rats [298,299]. Altogether, GPx is a relevant target for therapeutic approach towards neuropathic pain [300].

### 3.4. Thioredoxin

**Figure 16:**
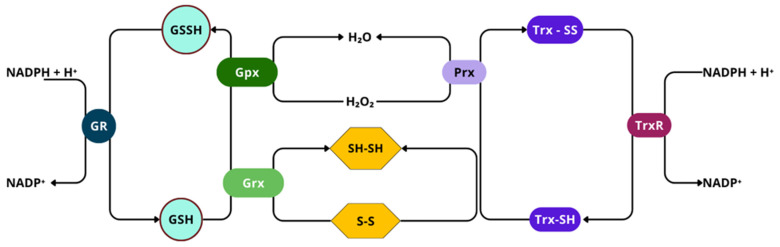
The thioredoxin system in neuronal antioxidant defense. Trx has the ability to catalyze the reductive conversion of disulfide (S-S) bonds into dithiol (-SH) bonds on substrates, due to its reductase power, acting through disulfide–dithiol bond exchange. The disulfide (S-S) bond in the oxidized Trx is reduced back to its original free thiol (-SH) group state, through the enzyme TrxR, using NADPH as an electron donor, ready to engage in the next catalytic cycle. GPx catalyzes the reduction of hydrogen peroxide (H_2_O_2_) to water. To do this, GPx utilizes glutathione (GSH) as an electron donor. Here, two molecules of GSH are oxidized to form a disulfide bond (S-S), resulting in GSSG (oxidized glutathione). Now, the oxidized glutathione (GSSG) is recycled back to its reduced form (GSH) to maintain its antioxidant capacity. GSSH: oxidized glutathione; GSH: glutathione; Gpx: glutathione peroxidase; Grx: glutathione reductase; GR: glutathione reductase; Trx: thioredoxine; TrxR: thioredoxine reductase; SH: dithiol bonds; SS: disulfide bonds.

The thioredoxin (Trx) system is formed by two oxidoreductase enzymes, Trx and TrxR, with NADPH as an electron donor. Trx are highly conserved across living cells, including mammals. The Trx system has greatly important functions protecting cells against oxidative stress and damage, being particularly important in the CNS cells, where it assists correct protein synthesis and the modulation of apoptosis [301]. Trx serves as a redox homeostasis regulator through the modulation of signaling pathways, induction of transcription factors, and pro-survival mechanisms [302]. Trx is depicted with a Cys-Gly-Pro-Cys active site vital for protein disulfide reductase of enzymes like peroxidredoxins (Prxs), ribonucleotide reductase (RNRs), and methionine sulfoxide reductase (MSRs), as it serves as an oxidoreductase and an electron donor [303]. Trx has the ability to catalyze the reductive conversion of disulfide (S-S) bonds into dithiol (-SH) bonds on substrates, due to its reductase power, acting through disulfide–dithiol bonds exchange [263]. The whole process happens as follows: Trx binds to the protein substrate that contains a disulfide bond (S-S); then, one thiol (-SH) group of the N-terminal cysteine in the active site (Cys-Gly-Pro-Cys) of the Trx performs a nucleophilic attack on the disulfide (S-S) bond of the substrate by electron donation, yielding an intermolecular mixed S-S between the Trx and the substrate. Afterwards, the second thiol (-SH) group in the C-terminal cysteine in the active site (Cys-Gly-Pro-Cys) of the Trx attacks the intermolecular mixed S-S bond, releasing the reduced substrate and leading to the creation of a disulfide (S-S) bond in the Trx itself. Finally, the disulfide (S-S) bond in the oxidized Trx is reduced back to its original free thiol (-SH) groups state, through the enzyme TrxR, using NADPH as an electron donor, ready to engage in the next catalytic cycle (Figure 16) [304].

**Figure 17:**
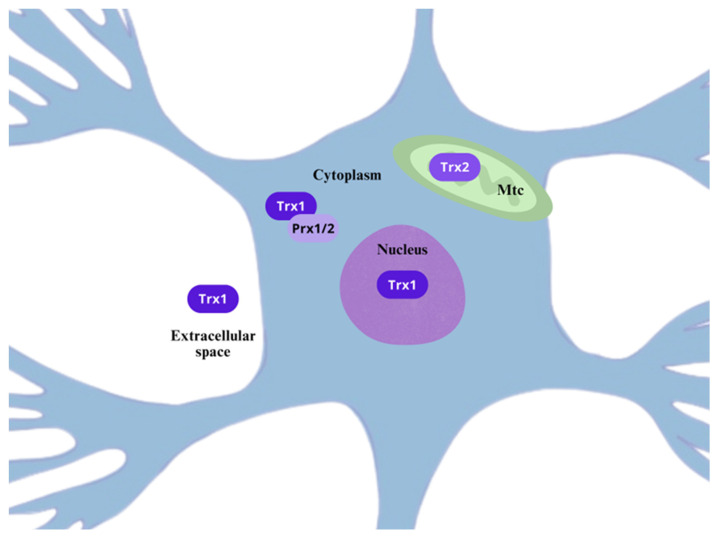
Glutathione peroxidase (Gpx) sites of expression in neuronal cell. Gpx drives the enzymatic reduction of H_2_O_2_ and hydroperoxides.

In mammals, there are two primaries strict Trx proteins: Trx1 and Trx2. Trx1 is the most widely studied and present mainly in the cytosol but can be found in the nucleus upon oxidative/nitrosative stress [305] or released through the cellular membrane [306]; Trx1 is coupled to Prx1 and Prx2, and MSR, being critical for cellular growth and apoptosis, and is expressed in neuronal cells (Figure 17). Thus, neurons with dysfunctional mitochondria induced by complex IV inhibition show increased H_2_O_2_ vulnerability due to low Trx expression [307]. The Trx2 isoform is localized in the mitochondria and is exceptionally relevant for maintaining mitochondrial redox balance, as well as for the protection of mitochondrial DNA and proteins from oxidative damage [308]. Similarly to Trx1, Trx2 is expressed in neurons, and shows lower expression levels after complex IV inhibition, making the cells more susceptible to H_2_O_2_ attack [307]. Both Trx1 and Trx2 are primarily expressed in sensitive neurons in the DRG and in the spinal cord and sciatic nerve and are particularly altered in nociceptive signaling sites after sciatic nerve injury, participating in the regulation of oxidative stress and redox homeostasis, relevant in the pathophysiology of neuropathic pain [309].

The above-mentioned antioxidant enzymatic proteins play critical roles as regulators of various redox signaling pathways, which profoundly depend on reversible tissue- and target-specific modifications of main thiols in proteins. The CNS, a highly oxidizing environment, is acknowledged as very vulnerable to reactive species effects carried out by nitrosative or oxidative damages. While each enzyme has a specific role, they work together through synergistic interactions, redundancy, and compartmentalization to ensure a robust, correct defense against reactive species and to guarantee cellular redox equilibrium.

## 4. Nitroxidative Stress in Neuronal Cells

Pain can be considered, in a wide sense, as a protective mechanism to alert to actual and imminent danger to tissue integrity, or as a warning to potential lesions, eliciting a myriad of responses across emotional, sensory, social, cellular, and molecular domains. When the sensory process that provides triggering signals for pain occurs, nociception initiates, comprising the detection of harmful or potentially harmful stimuli by nociceptors, as a normal response to such stimuli. That is, after nerve injury, the activation of macrophages, circulating neutrophils, and T cell recruitment to the site of the lesion, along with cytokines, chemokines, neurotransmitters, and ROS production, prompts peripheral and central sensitization [310]. In some cases of injury, it often leads to abnormal sensory phenotype expression in the nervous system, causing hypersensitivity due to peripheral nerves, dorsal root ganglia (DRG), or central nervous system damage, ultimately leading to neuropathic pain [311], which is defined as a pain caused by a lesion or a disease of the somatosensory system [312] estimated to affect approximately 7–10% of the global population [313], characterized by paresthesia, dysesthesia (tingling, numbness, pins, and needles sensation), associated with non- painful sensory neurological deficits in the disturbed area, together with motor and cognitive deficits, depending on the affected region [314].

Glial cells are important for homeostasis maintenance in the CNS, providing structural and nutritional assistance for neurons through hydro-electrolyte balance and neuronal synapsis (astrocytes), or surveying and protecting the neuronal milieu against potential disruptions of homeostasis and astrocyte activation (microglia) [315]. Immune cells of the CNS play a critical role in the development and continuance of neuropathic pain, as nerve insult leads to the neuroinflammation response, characterized by increased local vascular permeability, the activation of glial cells and inflammatory mediator release, enhancing pain sensitization [316]. Once microglia are activated, phenotypical and functional modifications occur, leading to proinflammatory cytokine release, such as TNF-α, IL-1β, and IL-6, cluster differentiation molecule 11b (CD11b), CD68, and ionizing calcium binder adapter molecule (Iba1) [9,317], all of which contribute in the early phase of neuropathic pain stablishment, and also causing astrocyte activation, supported by an increased expression of glial fibrillary acidic protein (GFAP) and CD11b, indicating a shift in astrocyte function, which in turn activates microglia, thus, creating cross-talk for neuropathic pain pathophysiology [194,318]. While microglia contribute initially to the establishment of neuropathic pain via the inflammatory response initiation, astrocytes are accountable for the long-term maintenance of such state [319,320].

Activated glia release chemokines, RONS, and neurotransmitters that modulate inhibitory and excitatory synaptic transmission, relevant for pain amplification [5]. Glutamate, the primary excitatory neurotransmitter is upregulated in astrocytes in response to inflammatory signals, contributing to central sensitization and pain amplification; aspartate, similarly to glutamate can be influenced by glial activation, contributing to excitatory signaling in neuropathic pain; and (Gamma-amynobutyric acid) GABA, the primary inhibitory neurotransmitter, responsible for reducing neuronal excitability and neuronal firing, can be downregulated in neuropathic pain, causing diminished inhibitory signaling, and consequently, increasing pain sensitization [316]. Additionally, purinergic receptors (P2X and P2Y), TLRs, Src kinase, and MAPK (JNK, ERK, and P38) pathways can be upregulated in conditions of nerve injury. This leads to increased expression of transcription factors in activated glia through various mechanisms, including further GF, proinflammatory cytokine and chemokine, and RONS production, contributing to pain sensitization, as described above [321]. In this sense, RONS act as redox sensors for signal transduction pathways modulating the neuroinflammatory process in neuropathic pain [37]. In case the prompting offense to glial cells is not resolved, chronic activation arises, reflecting in a continuum of glial activation states [322].

The molecular mechanisms underlying the neuropathic pain shift the microglial phenotype from a resting, small, highly branched, compact cell body and surveillance state to an activated bigger cell body, with a less branched, and ameboid state, causing an altered expression of cell surface proteins, growth factors, intracellular signaling molecules, and redox state consistent with inflammatory mediators release, such as cytokines and proteases contributing to the development of the neuroinflammation and neuropathic pain sensitization [311].

Morphologically, three states are described in the microglial cells, similar to that used in peripheral macrophages: M0 phenotype for the resting state, M1 phenotype for the classical activation state, and M2 phenotype for the alternative activation state [323]. The first state is characterized by the expression of genes related to neuronal function and development, whereas M1 and M2 phenotypes are induced by CNS insults and chronic pain, portrayed as mixed temporally changing and lacking equilibrium in the activation continuum. Thus, the M1 state is thought to lead to excessive RONS production and neuroinflammation, and M2 hampers an adequate immune response [323]. Also, astrocytes express antioxidant proteins to maintain overall redox homeostasis in the CNS under normal or pathological conditions [324,325]. In this sense, microglia and astrocytes can influence direct structural and molecular modifications in neuronal synapses, neuron differentiation, and the axon growth of mature neuronal cells, both physiologically, as part of normal CNS functioning, and pathologically to cause oxidative and nitrosative damage and an imbalance of redox signaling [308].

Microglial cells are of prominent relevance regarding the inflammatory process typical of neuropathic pain [326], as they release a large number of neuroactive substances, which in turn contribute to the RONS production. Among these RONS, it can be pointed out that Nox- and iNOS-derived radicals in neurons, such as O•− and NO, contribute to the cell death of dopaminergic neurons in neuropathic pain [17,114]. Microglia under oxidative stress produce increased inflammatory mediators that can move through the cell membrane, working as signaling molecules to other cells or inducing peroxynitrite formation causing DNA fragmentation, lipid oxidation, and neuronal death in a faster manner than that perceived in astrocytes [327]. In microglia cells producing O•− catalyzed by nitrate and nitrites, O_2_ and H_2_O_2_ levels are rapidly imbalanced, disturbing microglial functions, which in turn facilitates adjacent neuronal cell damage induced by ROS [328]. Elevated levels of RONS have been proved to increase proinflammatory cytokines in microglial cells, whereas lower RONS levels are associated with anti-inflammatory cytokine upregulation [114]. ROS generated by Nox2 contributes to neuropathic pain inducing the activated microglia phenotype, further enhancing the development of the chronic glial activation state in the spinal cord, and assisting the interaction of neurons and macrophages in the DRG [112,329,330], pointing the central and peripheral role in neuropathic pain, whereas Nox4’s role is primarily related to peripheral demyelination, causing peripheral nerve sensitization, and therefore adding up to the overall effect of neuropathic pain [114,116].

In addition to microglial cells, other CNS cells can produce O•− and NO wherever NOS and Nox are expressed, contributing to radical-mediated damage, as seen in β-amyloid-induced neurotoxicity in astrocytes [331]. Astrocytes serve key functions in neuronal recovery but can, under certain circumstances, be neurotoxic. When activated, they also release NO, TNF, and ROS to the extracellular space, which can negatively affect axon regeneration and nerve development, and lead to neuronal injury and death through mitochondrial stress, DNA strand breaks, and lipid peroxidation [332]. Moreover, when astrocytes suffers sustained oxidative stress, glutamate transporters, astrocyte–neuron communicating enzymes [333], and connexins undergo substantial modifications, which are implicated in the establishment of neuropathic pain [334]. Mitochondria dysfunction, NADPH-derived ROS, and RNS in astrocytes are the main factors accountable for oxidative and nitrosative stress, which is distributed across the long and thin processes and cell bodies, thus leading to astrocyte degeneration, SOD1 aggregation, and ischemic/reperfusion injuries [335,336].

In a 1-methyl-4-phenyl-1,2,3,6-tetrahydropyridine (MPTP) model of cell death of dopaminergic neurons, researchers aimed to document whether chronic exposure to small doses of MPTP were capable of affecting neurons from the dorsal horn of the spinal cord, responsible for the sensory component of pain. Considering that MPTP inhibits mitochondrial complex I, this leads to an augmented production of ROS, oxidative stress, lipoperoxidation, and mitochondrial membrane rupture, leading to apoptosis pathways and neuronal damage, evidenced by decreased calbindin D28K, calretinin, and parvabulmin-positive neurons in the laminae II; a reduction of SP interneurons and Met—enkephalin-positive fibers in laminae I and II; and finally, increased alpha-synuclein in spared neurons and fibers, establishing a clear connection between mitochondrial dysfunction, increased ROS production, and neuronal damage [337]. Also, Nox-deficient animals in an inflammatory model of dopaminergic cells death were found to be less affected than the wild type of animals, suggesting a direct role of Nox-derived O•−in the mechanisms of microglial cell activation and neurotoxicity [338].

Furthermore, TRPA1, TRPM2, and TRPV1 are activated by nitroxidative species, mediating the integration of endogenous and exogenous sensory stimuli, at the peripheral and central terminals and somas of afferent neurons [37]. TRPA1 and TRPV1 are expressed by peptidergic C fibers, activated directly by peroxidized and nitrated phospholipids and carbonylated proteins, as well as aldehyde formation, due to oxidative stress [82,339]; TRPA1 is primarily related to nociceptive hypersensitivity development, while TRPV1 is crucial for nociceptive hypersensitivity and thermal hyperalgesia [82]. TRPM2 is expressed in neurons and microglia, activated directly via H_2_O_2_ and cytosolic ADP—ribose [340,341], which are by-products of mitochondrial damage and are associated with spinal cord microglia activation, the local migration of macrophages after nerve injury, proinflammatory response, and promoting nociceptive hypersensitivity and are also crucial in the activation process of the MAPK pathway, causing NF-κB nuclear translocation, consequently increasing the proinflammatory cytokine and chemokine production [340,342]. In this context, TRP plays a critical role in the development and maintenance of neuropathic pain through polymodal activation, including chemical, thermal, and mechanical signals, inducing ionic changes across neuronal membranes, and initiating downstream signaling pathways comprising: AMPK, MAPK, NF-κB, and TGF- β. TRP also enhance hyperexcitability via the increased expression and activity of TRP, leading to spontaneous pain and disturbed sensitivity [343]. Additionally, such a state of increased expression and activity of TRP leads to the hyperexcitability, apoptosis, hyperactivation, and infiltration of immune cells, amplified levels of proinflammatory cytokines, mitochondria dysfunction, and autophagia as seen in models of diabetic neuropathy in peripheral nerves [344], cancer-induced pain [345], chemotherapy-induced peripheral pain [346], and trigeminal neuropathic pain [22]

It is widely known that proteins can undergo reversible modifications through oxidative actions in amino acids. Amongst the most redox-sensitive amino acids it can be cited that the -SH group cysteine, and the aromatic ring of tyrosine [21], displaying different predispositions to endure RONS alterations depending on the specific oxidants in the redox reaction, the proportion and distribution of thiol groups, transition metal ion presence as prosthetic groups, and motifs and amino acid residue exposure on the molecular surface of the proteins, as well as different levels of chemical modifications ranging from reversible to irreversible, and specific or non-specific union, which depends on the selective attachment to residues and their location, or multiple sidechain and backbone sites affected, respectively [347].

**Figure 18:**
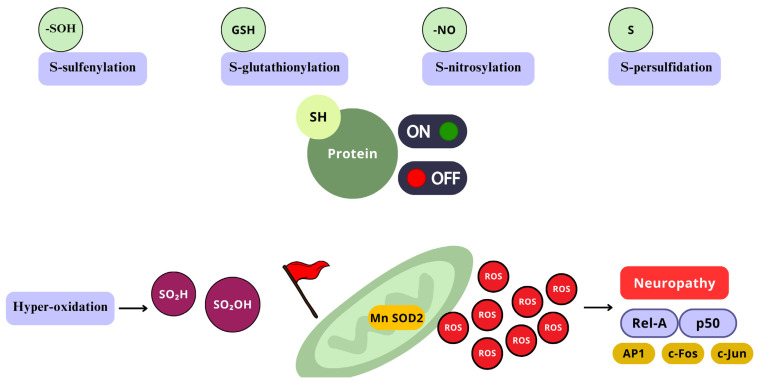
Reversible and irreversible proteins change under ROS/RNS. Reactive species serve as redox-sensitive switch molecules due to amino acid -SH group cysteine, or to aromatic rings of tyrosine, for modulating protein activity. Nevertheless, when RS• interact with NO to form S-nitrosylated cysteine residues in proteins, or undergo hyper-oxidation, to produce sulfinic and sulfonic cysteine after interaction with H_2_O_2_, ONOO^−^, and HOCl, changes can become irreversible, impairing antioxidant defense and leading to neuronal damage.

From a redox perspective, the reversible and specific oxidation of a few or at least one single redox-sensitive residue in a protein by an electrophile molecule can be enough to induce chemical modification in a switch manner for modulating the protein activity [348]. Some of the plausible amino acid residue modifications bearing redox switch capacities can be elicited through cysteine residue S-sulfenylation, S-glutathionylation, S-nitrosylation, and S-persulfidation, given that cysteine residues are key redox-sensitive molecules due to the presence of -SH groups, bestowing structural and functional flexibility to proteins (Figure 18) [349,350].

Cysteine residues are usually found in catalytic and regulatory sites of enzymes, signaling pathway effectors, and transcription factors, and they can initially undergo the ionization to thiolate (R-S^−^) which is the deprotonated form of -SH, and further undergo one- or two-electron oxidations, to yield thiyl radicals (RS•), or cysteine sulfenic acid (-SOH), respectively, both reversible. Then, RS• can interact with NO to form S-nitrosylated cysteine residues in proteins, or undergo hyper-oxidation, to produce sulfinic and sulfonic cysteine after interaction with H_2_O_2_, ONOO^−^, and HOCl, which are irreversible (Figure 18) [351], as seen in the irreversible oxidation of SOD1 Cys111 to its sulfinic and sulfonic states, present in ALS mice models that presented signs of peripheral pain [352,353,354]. Accordingly, in spite of ALS being a neurodegenerative disease characterized by upper and lower motor neuron degeneration, it has been recently found to have a sensory neuropathy component observed in up to 20% of ALS human patients, pain among them, affecting unmyelinated small and myelinated large fibers as part of the process of primary neurodegeneration [355,356], potentially leading to neuropathic pain, and relevant to understand the full spectrum of ALS, associated with SOD1 mutation. Furthermore, cysteine residues of proteins and peptides exposed to NO and ONOO^−^ can suffer S-nitrosylation (-SNO groups formation) and S-glutathionylation (SS and GSH mixed groups formation); such reactions are relevant to cell signaling, protein stability and activity, and redox homeostasis through GST, Grx, and Trx [357].

Tyrosine residues oxidation leading to 3–hydroxytyrosine, 3-nitrotyrosine, halogenated tyrosine, and intramolecular tyrosine cross-links are deeply associated with interference with cellular functions, as seen in endogenous ROS exposure, yielding inter- and intramolecular cross-links in proteins, accompanied by spontaneous fragmentation of the polypeptide chains involved; some of these cross-links, like 3,3′-dityrosine, are biomarkers of oxidative stress, as seen in a study using a neuropathic pain model of chronic constriction injury, where they found dityrosine-containing protein cross-links exposition to activate satellite glial cells (SGCs) in the DRG, and a subsequent increase in proinflammatory mediators and ROS, mediated by the NF-κB pathway and the receptors for advanced glycation end products, exacerbating hyperalgesia [358]. As for tyrosine and tryptophane nitration, once they react with OH•, and subsequently form tyrosyl and tryptophanyl radicals, both can further react with NO_2_• after covalent attachment to produce 3-nitrotyrosine and 6-nitrotryptophan, respectively [359].

Proteotoxic stress has been linked to neuropathic pain through nitration reactions, which profoundly trigger chemical and structural changes in proteins, leading to function alterations and cell signaling modulation [36,99]; as such, peroxynitrite, a cytotoxic pro-inflammatory and pronociceptive agent is portrayed as a main factor in the development of peripheral and central sensitization associated with neuropathic pain [360,361]. The mitochondrial MnSOD can be negatively affected by nitration, rendering it inactive, and thus contributing to the perpetuation of a vicious cycle of free radical formation. This, in turn, leads to further nitrosative damage to relevant enzymes unable to fight back against the development and maintenance of hyperalgesia and central sensitization [36]. The accumulation of ROS and RNS, increased during oxidative and nitrosative stress conditions, can induce the activation of transcription factors such as AP-1, NF-κB, and MAPKs, which can activate and increase the expression and production of COX enzymes and PGs [362,363].

Whenever antioxidant systems are exceeded by the production of ROS or RNS, the outcome is likely to cause an oxidative or nitrosative stress state, in which subsequent damage to biomolecules like proteins, membrane lipids, and nucleic acids leads to cellular and tissue dysfunction and cell death via necrosis, apoptosis, and autophagy, implicated in chronic pain of neuropathic origin [364]. Nevertheless, the final outcome or the biological impact of the cross-links between antioxidants and pro-oxidants will depend on the specific type interacting, physicochemical properties, cellular and subcellular location and gradient, the rate of formation and degradation, and quantity. Thus, in recent years, not only antioxidants, but also RONS have gained more attention from the scientific community, as the regulatory roles of RONS have emerged as adaptive mechanisms involved in cellular homeostasis, in low steady concentrations, for reversible redox-sensitive modifications in functional motifs of several proteins related to cell signaling.

## 5. MnPs: Mechanisms of Action and Potential Therapy for Neuropathic Pain

The role of oxidative and nitrosative stress has been stablished for chronic pain of neuropathic origin, through the effects of hyper-physiological levels of reactive species on neuronal and glial cells [365]. Antioxidant therapies from diverse origins, aiming for the restoration of normal cell functioning, have been applied for various chronic pain disorders, with different, sometimes conflicting, and specific pain type-dependent outcomes, either positive, negative, or not clear [366,367]. Among the most studied synthetic therapeutic antioxidant compounds, there is a group of proteins named SOD mimics. By designation, SOD mimics are compounds with the ability to catalyze the oxidation and reduction of O•−. Yet, it has been proved that in addition to reaction with O•−, SOD mimics can also catalyze redox reactions with H_2_O_2_, ONOO^−^, thiols, and others under specific conditions [368], working as oxidants and reductants.

More specifically, there is a class of metalloproteins of low molecular weights with a central metal ion of manganese contained in the porphyrin ring of synthetic origin, exhibiting different oxidation states of manganese Mn(II), Mn(III), and Mn(IV), which contributes to their redox applications, and these are named manganese porphyrins (MnPs) [369]. MnPs were initially, in a very narrow way, seen as simply scavengers of O•−, but the new knowledge about redox chemistry applied to cellular physiology proved its worth based on evidence of their potential role as therapeutics, due to their physicochemical properties, longer half-life, lower cost of production compared to SOD enzymes, and lack of immunogenic reactions in biological systems, hence, affecting signaling pathways and cellular functions such as proliferation, differentiation, and cell death [26], through the modulation of the redoxome [370].

In addition to the specific reactive species scavenged by MnPs, even more importantly, there are other aspects intrinsic to their structure that must be taken into consideration when applying MnSOD mimic therapeutic strategies for neuropathic pain. First, the half-wave redox potential (E_1/2_(O•−)), which determines whether a compound can be readily oxidized and/or reduced by the substrate within the redox active molecular targets in the cell. E_1/2_(O•−) is a reliable measure of physiological relevance, as it compares the electrochemical potency at half of the wave of the compound to that of the SOD enzyme (+300 mV vs. normal hydrogen electrode—NHE) in relation to O•−; the closer it is, the more biologically compatible the compound is for electron interchange among reactive species and signaling proteins in the cellular context. Potentials that are too high or too low can be toxic or inefficient, respectively [368]. A MnP within a compatible range will also allow a more specific action of the compound over the target molecules, without interfering with other biological processes, and more stability, avoiding fast degradation or inactivation [371].

Secondly, the intrinsic catalytic activity of the SOD mimic protein (Kcat) for the catalysis of O•− is another reliable measure of the therapeutic potential of the compound, as it quantifies the efficiency to catalyze the redox reaction, expressing the number of substrate molecules converted into the reaction product for each molecule of the compound by the unit of time, when the compound is fully saturated with the substrate (i.e., SOD Kcat(O•−) ~2 × 109 M^−1^·s^−1^) [372]. Such factors establish not only the catalytic efficiency, but the rate of the reaction, which is relevant for reaching the therapeutic outcomes in a reasonable time, and the compatibility with the cellular redox conditions, that is the pH, temperature of the reaction, and substrate concentrations [373]. Notwithstanding these factors, the in vivo efficacy of metalloporphyrin-based SOD mimics is not only associated with their SOD-like activity, but with other factors like the shape, size, and bulkiness of the molecule [374], bioavailability, biodistribution in different cell kinds and subcellular spaces, and reactivity toward other cellular biomolecules [375], which will dictate the overall therapeutic effect of the treatment. Assuming the rich environment of redox-based signaling processes occurring in the cellular metabolism through cellular respiration, glycolysis, cyt P450 detoxifying system, ETC, NO synthesis, and others, an approach targeting these redox-signaling pathways for therapeutic purpose would make more sense when assessing the effects of MnP on redoxome alterations, than on separate scavenging activity toward specific reactive species [23].

The main consideration is that, under impaired conditions of neurons’ ability to bear peroxides elimination, the excessive accumulation of H_2_O_2_ as a byproduct of the O•− dismutation through MnP might reach toxic levels. That is, the overall antioxidant power of the synthetic enzyme will only be efficient if H_2_O_2_ is also removed [368]. Cationic MnPs are not potent scavengers of H_2_O_2_, but once reduced in vivo, may bind O_2_ to O_2_•− and H_2_O_2_ [376]. Yet, cationic MnPs have higher Kcat(O•−) in comparison to neutral or anionic MnP, since O•− is negatively charged; therefore, they are prone to be attracted to the positively charged cationic MnPs, facilitating a more efficient interaction and better catalytic activity. This translates into a more effective MnP in the detoxification and utilization of the O•− radical for antioxidant mechanisms [377,378]. In addition, when MnP holds a highly positive reduction potential, it is very likely that they will tend to accept electrons by cellular reductants, rather than give electrons away; in this process, Mn will be reduced to Mn(II), which will reduce O•− to H_2_O_2_, working as superoxide reductase, rather than superoxide dismutase [379].

The electrophilic nature of MnP, especially Mn(III) explains its tendency to react with or bind to anionic substrates, normally electron-rich, such as HOO^−^, RS^−^, ClO^−^, ONOO^−^, etc. When MnP enters the cell, it can straightaway suffer reduction while inducing the oxidation of thiols, thus, working as an oxidant and antioxidant [368]. As proved by previous research, MnPs can work as suppressors of glycolysis and mitochondrial respiration [379], exhibit significant antihyperglycemic activity, and enhance neuronal death [380]; additionally, MnP can induce NF-κB inactivation [381] and protective responses against oxidative damage to neuronal and glial cells [248,365], as well as reduced NF-κB expression, thus, resulting in a significative decrease in proinflammatory cytokines such as IL-1β and TNF-α [382].

MnPs with potent log Kcat(O•−) are also potent ONOO^−^ scavengers. As previously stated, electron-deficient MnPs react or bind to electron-rich anionic substrates, such as ONOO^−^. When Mn(III) is oxidized to Mn(IV), either through an O•− or ONOO^−^ reaction, reducing itself back to Mn(III) with cellular reductants present in the electron pool, yielding the highly oxidizing radical nitrogen dioxide (NO•_2_), or through a two-electron approach to Mn(II) yielding nitrite (NO_2_^−^), this latter reaction being the most likely to occur in cells, as MnPs are maintained in the Mn(II) state by cellular reductants [383]. In addition, MnPs can interact with deprotonated reductants like GSH, cysteine, and protein thiols [26]; in this sense, the MnP works as an oxidant, accepting electrons from the substrates, and being reduced to either the Mn(III) or Mn(II) lower oxidation states as a result, while oxidizing the reductants.

A significant degree of interaction between MnP and thiols has been reported, specifically towards cysteine residues present in the p50 subunit of the NF-κB protein, resulting in disulfide formation [384]. The S-glutathionylation of the p65 NF-κB subunit, which is responsible for the κB attachment to certain DNA regions, thereby initiating the transcription of target genes, has been demonstrated in hyaluronan degradation-induced inflammation in mice [385]. In such cases, the MnP functioned as an oxidant, yielding a GS radical, which combined with additional GS to produce GSSG radicals; GSSG, when oxidized by O_2_, produces O•−. However, glutathionylation will only be significant if GSH and H_2_O_2_ are present, and GSSG can be safely removed if glutathione reductase (GR) is present in sufficient levels [381]. The antioxidative effect of MnP, as previously mentioned is only effective when physiological levels of peroxide enzymes are present to remove H_2_O_2_. Hence, the cycling of MnP with GSH or cysteine would lead to an accumulation of H_2_O_2_, contributing to the further oxidation of biomolecules of significant importance in the homeostasis complex process [368]. In a chemotherapy-induced neuropathic pain model in rats, mitotoxicity influenced by reduced ATP production in mitochondria due to respiratory complex I and II inhibition in primary nerve sensory axons was thwarted by the administration of Mn(III) 5,10,15,20-tetrakis(N-n-hexylpyridinium-2-yl)porphyrin (MnTE-2-PyP(5+)), without compromising anti-tumor effect, and mechanical hypersensitivity was reversed [386].

Although evidence for manganese-based porphyrins’ therapeutic effects over neuropathic pain is scarce, acknowledged evidence of iron-based porphyrins exists [387,388]. Manganese and iron porphyrins can both dismutate superoxide anion, although their efficiency and pathway might differ in favor of manganese porphyrins, which have an overall mechanism more promising regarding interaction with cellular reductants and flavoenzymes, and the production of •OH after interacting with H_2_O_2_, as MnPs have a very limited capacity to interact with H_2_O_2_, contrary to iron porphyrins, which can produce •OH in a Fenton-like reaction, contributing to reactive species production in the cell [28,374]. Yet, it is important to acknowledge that iron porphyrin complexes perform remarkable peroxynitrite decomposition catalyst activity, highlighting the potential of porphyrin-based research for therapies in the context of neuropathic pain.

Structural and functional neuropathic alterations in diabetic type 2 rats were evaluated and the therapeutic potential of peroxynitrite decomposition catalysts Fe(III) tetrakis-2-(N-triethylene glycol monomethyl ether)-pyridyl porphyrin (FP15) and Fe(III) tetra-mesitylporphyrin octasulfonate (FeTMPS) were assessed. Mechanical and sensorial deficits and thermal allodynia, as well as nitrotyrosine and poly(ADP-ribose) levels were restored with both treatments, compared to untreated animals [389]. Ischemia/reperfusion-induced neuropathic pain has also been evaluated regarding the peroxynitrite decomposition catalyst effect of intraperitoneal FeTMPyP [5,10,15,20-tetrakis (N-methyl-4′-pyridyl)porphyrinato iron (III)]. A significant enhancement of mechanical withdrawal threshold and phosphorylated NMDA subunit 1 were observed in treated animals, consequently reducing mechanical allodynia and central sensitization [390]. The anti-nociceptive and antioxidant effect of peroxynitrite decomposition catalyst FeTMPyP was tested in chronic constriction of the sciatic nerve-induced neuropathic pain in rats. High iNOS, NF-κB, IL-6, and TNF-α expression levels in sciatic nerves, together with increased poly (ADP) ribose DRG levels were completely reversed after treatment, reducing PARP overactivation, oxidative stress, and normalizing behavioral and functional pain parameters [391].

## 6. Final Considerations

This review evaluated the role of reactive species in cell signaling, focusing on their redox-sensitive characteristics and their link to neuropathic pain. Reactive oxygen and nitrogen species, significant for cellular signaling pathways, act as both mediators of cellular damage and modulators of physiological processes. Their dysregulation, especially under oxidative stress, has been increasingly linked to the sensitization of pain pathways, highlighting the potential of targeting redox-sensitive mechanisms for novel therapeutic strategies. Enzymatic antioxidant systems play a crucial role in maintaining redox homeostasis. Enhancing these defenses offers a promising strategy to reduce oxidative and nitrosative damage associated with neuropathic pain. Among emerging therapeutic agents, manganese porphyrins (MnPs) stand out due to their mimetic antioxidant properties, offering significant potential for addressing redox imbalances and neuropathic pain. This review underscores the significance of understanding the intricate dynamics of reactive species, subcellular compartment gradients, and MnP-mediated redox chemistry in the context of neuronal dysfunction. Further research is essential to optimize the design of biocompatible, target-specific MnP molecules and to elucidate their safety and efficacy in clinical settings. Advancing knowledge in this field may pave the way for innovative treatments for neuropathic pain, ultimately improving the quality of life for affected individuals.

## Data Availability

Data will be made available on request.

## Abbreviations

AP-1	Activator Protein 1
ATF6	Activator Transcription Factor
BDNF	brain-derived neurotrophic factor
CAT	catalase
CHOP	C/EBP Homologous Protein
Cys	cysteine
EGF	epidermal growth factor
eNOS	Endothelial Nitric Oxide Synthase
ER	endoplasmic reticulum
ERK	Extracellular Signal-Regulated Kinase
ERO-1	Endoplasmic Reticulum Oxidoreductin-1
ETC	electron transfer chain
FAD	flavin adenine dinucleotide
GH	glutathione
GLUT1	Glucose Transporter 1
GPx	glutathione peroxidase
GR	glutathione reductase
GRP78	Glucose-Regulated Protein 78
GSH	reduced glutathione
GSSG	oxidized glutathione
H_2_O_2_	hydrogen peroxide
HIF-1α	Hypoxia-Inducible Factor 1α
HO•	hydroxyl radical
HOCL	hypochlorous acid
HOO•	hydroperoxyl radical
IL	interleukin
iNOS	Inducible Nitric Oxide Synthase
IRE-1α	Inositol-Requiring Kinase 1α
IRI	ischemic reperfusion injury
JNK	c-Jun NH2 quinase terminal
KEAP1	Kelch-like ECH-associated protein 1
LOOH	lipidic peroxides
M	Molar
MAPK	Mitogen-Activated Protein Kinase
MCU	Mitochondrial Calcium Uniporter
MDA	malondialdehyde
Met	methionine
MnP	manganese porphyrin
mtNOS	Mitochondrial Nitric Oxide Synthase
NAD	nicotinamide adenine dinucleotide
NCX	Na^+^/Ca^2+^ exchangers
NF-κB	Nuclear Factor kB
NGF	nerve growth Factor
nNOS	Neuronal Nitric Oxide Synthase
NO	nitric oxide
NOX	NADPH oxidase
NRF2	Nuclear factor (erythroid-derived 2)-like 2
O_2_•−	superoxide radical anion
ONOO^−^	peroxynitrite
PDI	Protein Disulfide Isomerase
PERK	Protein Kinase (PKR)-Like Endoplasmic Reticulum Kinase
PG	prostaglandin
PI3K	phosphatidylinositol 3 kinase
PIP	phosphatidylinositol phosphate
PIP2	phosphatidylinositol biphosphate
PIP3	phosphatidylinositol triphosphate
PMCA	the plasma membrane calcium ATPase
PPP	pentose phosphate pathway
RNS	reactive nitrogen species
ROO•	peroxyl radical
ROS	reactive oxygen species
RS•	thiyl radical
SERCA	Endoplasmic Reticulum Calcium ATPase
SOD	superoxide dismutase
Tyr	tyrosine
VGF	vascular growth factor
XO	xanthine oxidase
